# Transcriptomic and Metabolomic Profiling in *Helicobacter pylori–*Induced Gastric Cancer Identified Prognosis- and Immunotherapy-Relevant Gene Signatures

**DOI:** 10.3389/fcell.2021.769409

**Published:** 2021-12-24

**Authors:** Duanrui Liu, Jingyu Zhu, Xiaoli Ma, Lulu Zhang, Yufei Wu, Wenshuai Zhu, Yuanxin Xing, Yanfei Jia, Yunshan Wang

**Affiliations:** ^1^ Research Center of Basic Medicine, Jinan Central Hospital, Cheeloo College of Medicine, Shandong University, Jinan, China; ^2^ Research Center of Basic Medicine, Jinan Central Hospital, Shandong First Medical University, Jinan, China; ^3^ Department of Gastroenterology, Jinan Central Hospital, Shandong First Medical University, Jinan, China

**Keywords:** *Helicobacter pylori*, gastric cancer, cancer metabolism, tumor environment, prognosis

## Abstract

**Background:** Chronic *Helicobacter pylori* (HP) infection is considered the major cause of non-cardia gastric cancer (GC). However, how HP infection influences the metabolism and further regulates the progression of GC remains unknown.

**Methods:** We comprehensively evaluated the metabolic pattern of HP-positive (HP+) GC samples using transcriptomic data and correlated these patterns with tumor microenvironment (TME)–infiltrating characteristics. The metabolic score was constructed to quantify metabolic patterns of individual tumors using principal component analysis (PCA) algorithms. The expression alterations of key metabolism-related genes (MRGs) and downstream metabolites were validated by PCR and untargeted metabolomics analysis.

**Results:** Two distinct metabolic patterns and differential metabolic scores were identified in HP+ GC, which had various biological pathways in common and were associated with clinical outcomes. TME-infiltrating profiles under both patterns were highly consistent with the immunophenotype. Furthermore, the analysis indicated that a low metabolic score was correlated with an increased EMT subtype, immunosuppression status, and worse survival. Importantly, we identified that the expression of five MRGs, GSS, GMPPA, OGDH, SGPP2, and PIK3CA, was remarkably correlated with HP infection, patient survival, and therapy response. Furthermore, the carbohydrate metabolism and citric acid may be downstream regulators of the function of metabolic genes in HP-induced GC.

**Conclusion:** Our findings suggest that there is cross talk between metabolism and immune promotion during HP infection. MRG-specific transcriptional alterations may serve as predictive biomarkers of survival outcomes and potential targets for treatment of patients with HP-induced GC.

## Introduction

Gastric cancer (GC) is the fifth most common and fourth most common cause of cancer-related death worldwide (Sung et al., 2021). Chronic *Helicobacter pylori* (HP) infection is considered the major cause of non-cardia gastric cancer, with almost all cases due to the presence of these bacteria (Plummer et al., 2015). However, despite the high significance of HP for human health, it seems that the molecular infection mechanisms and intracellular signaling pathways during colonization have not been fully elucidated. Therefore, the identification of new subtypes and functional pathways of HP-induced GC by recognizable molecular profiles and the exploration of novel key gene targets for monitoring GC progression are urgent requirements.

Host metabolic dysregulation in cancer pathogenesis is emerging as a key strategy for microbial remodeling of the infection microenvironment (Kim I.-J. et al., 2018). A growing body of evidence has demonstrated a strong connection between HP infection and metabolism. HP infection is associated with lipid and glucose metabolism (Gao et al., 2008; Satoh et al., 2010; Buzás, 2014). Changes in the expression of metabolism-related genes may underlie the molecular mechanism of cancer cell metabolic reprogramming. Zhou et al. revealed that HP infection promoted hepatic insulin resistance by affecting the c-Jun/miR-203/SOCS3 signaling pathway (Zhou et al., 2015). We and others have previously identified gene markers of HP+ GC that have prognostic significance (Liu et al., 2019; Liu et al., 2020). However, the impact of metabolism-related genes (MRGs) on prognosis and therapy in HP + GC has not been clearly defined.

Encouragingly, recent advances in the field of cancer immunotherapy, such as immune checkpoint inhibitors (ICIs), have led to new and powerful cancer management models. However, large numbers of patients do not respond to these clinically approved immunomodulatory drugs. In addition to immune mechanisms (e.g., recruitment of immunosuppressive cells or factors and impaired antigen presentation) that may lead to immune escape of tumor cells, increasing evidence supports the hypothesis that the dysregulation of energy metabolism could also contribute to the failure of cancer immunotherapy (Martinez-Outschoorn et al., 2017). Notably, metabolic enzymes and their products have become increasingly important as potential drug targets in recent years (Beger et al., 2016; DeBerardinis and Chandel, 2016). HP has been proposed to be involved in immune escape by limiting M1 macrophage activation and polyamine metabolism (Hardbower et al., 2016). It remains unknown whether there are metabolic interactions between immune cells and cancer cells in HP-induced GC.

In the present study, we comprehensively explored the expression of genes across key cellular metabolic pathways in HP*-*induced GC from GEO datasets. Subsequently, we confirmed two subgroups (Cluster 1 and Cluster 2) using consensus clustering for MRGs associated with HP infection that stratified the prognosis, different immune cell infiltration, and CTLA4 expression in GC patients. Next, we developed a methodology to quantify the metabolic pattern (a metabolic score). Importantly, we identified significantly high and low expression of multiple MRGs—GSS, GMPPA, OGDH, SGPP2, and PIK3CA—correlated with patient survival, chemotherapeutic drug sensitivity in HP-positive (HP+) GC, and ICI therapeutic response. We further validated that HP infection remarkably upregulated the expression of the five MRGs in MKN45 GC cells. Meanwhile, the downstream metabolite alteration and activated pathways were validated by metabolomics. These findings may contribute to a better understanding of the unique pathogenesis of HP*-*induced GC and allow for maximum efficacy of genetic, cellular, and immune therapies.

## Materials and Methods

### Data Collection and Processing

We downloaded level 3 mRNA expression, mutation, and clinical data from 375 GCs and 32 normal control samples from The Cancer Genome Atlas (TCGA, http://cancergenome.nih.gov/), and we derived GSE62254 from the Gene Expression Omnibus (GEO, https://www.ncbi.nlm.nih.gov/geo/). We obtained immune subtype information of the TCGA sample from UCSC Xena (http://xena.ucsc.edu/). Clinical information on GSE62254 was acquired from Cristescu et al. (2015). Additionally, 72 controls with HP− GC samples and 55 cases with HP+ GC were enrolled from GSE62254, which exploited the GPL570 platform (Affymetrix Human Genome U133 Plus 2.0 Array). The mRNA microarray dataset was normalized before being downloaded from the GEO database. The probe identifiers of the gene matrix file were converted to gene symbols according to the annotation file of the corresponding platform. The sum of gene expression values was processed by log2, and if multiple probes corresponded to the same gene symbol, the average value was used as the final expression value. The relevant data provided by TCGA and GEO are public and open; therefore, additional ethical approval was not required.

### Metabolism-Related Gene Extraction

In the present research, all MRGs originated from the Kyoto Encyclopedia of Genes and Genomes (KEGG) metabolism–related gene sets (“c2. cp.kegg.v7.2. symbols”; http://software.broadinstitute.org/gsea/downloads.jsp). By integrating the entire gene set of the samples with the metabolic gene set, 860 metabolism-related genes (Supplementary Table S1) were identified in the transcriptome data. Gene expression was also extracted from GSE62254 and analyzed.

### WGCNA Analysis

The expression file of GSE62254 was applied for the weighted gene co-expression network analysis (WGCNA) *via* WGCNA R package. We applied WGCNA to investigate the link between the clinical traits and expression modules (Langfelder and Horvath, 2008). At first, the gene pairs with Pearson coefficients above the threshold were fitted into a matrix. Next, the adjacent matrix was established by power function. The module detection of closely related gene clusters is performed once the weighted network is constructed. To evaluate the degree of gene association in the network and to minimize spurious connections among genes, topological overlap was inserted to discern the modules of highly similar genes. At last, similar gene expression patterns are grouped into the same color module. We used the Heatmap toolkit in R to calculate correlations between gene modules and clinical features and to plot the heatmap.

### Disease Connectivity Analysis and Consensus Clustering for HP-Associated MRGs

Diseases enriched with 44 HP-MRGs (Supplementary Table S1) were recognized by the Comparative Toxicogenomic Database (http://ctdbase.org/, Bonferroni-corrected *p* value<0.05) (Davis et al., 2019). To further explore the function of HP-MRGs, the HP+ GC patients were grouped into different clusters by ConsensusClusterPlus (resample rate of 80%, 50 iterations, and Pearson correlation, http://www.bioconductor.org/) on the basis of 44 HP-MRG expression (Wilkerson and Hayes, 2010). We used the consensus clustering algorithm to identify subgroups number and their stability. In addition, principal component analysis (PCA) was applied to explore the gene expression models in distinct HP+ GC groups.

### Tumor-Infiltrating Immune Cell Evaluation and ESTIMATE

We estimated the abundances of 22 distinct TIICs with the expression profile of HP+ GC by using the CIBERSORT algorithm (http://cibersort.stanford.edu/) (Newman et al., 2015), which is considered more suitable for analyzing the unknown mixture content and noise than the former deconvolution approach. In this research, the fraction of immune cells of GSE62254 samples was estimated using R package “CIBERSORT.” The Estimation of Stromal and Immune Cells in Malignant Tumors using Expression data (ESTIMATE) algorithm (Yoshihara et al., 2013), which makes use of gene expression signatures to predict the cellularity of the tumor and the purity of the tumor. We used the ESTIMATE algorithm *via* the R “estimate package” (Yoshihara et al., 2013) to determine the stromal scores, ESTIMATE scores, and immune scores for each sample included in GSE62254, respectively.

### Construction of the Metabolic Score

First, an unsupervised clustering method (K-means) (Wong and Hartigan, 1979) for categorizing the patients in the GSE62254 cohort as per HP-MRGs values was used. Furthermore, the random forest classification algorithm was used to conduct dimension reduction for reducing noise or redundant genes (Kursa and Rudnicki, 2010). Next, the clusterProfiler R package (Yu et al., 2012) was applied to annotate gene patterns. A consensus clustering algorithm (Monti et al., 2003) was adopted to identify the cluster of genes. Then we curated the expression profiles of the finalized genes (Supplementary Table S1) for the PCA and extracted principal component 1 as the signature score. This approach focuses mainly on the scores of well-correlated (or anticorrelated) genes with the largest blocks in the set, while reducing the contribution weight of genes that are not related to other pool members. Last, we applied an equation similar to the previous study (Sotiriou et al., 2006; Zeng et al., 2019) to define the metabolic score:

$$\mathrm{Metabolic}\;\mathrm{score}=\sum^{\text{​}}PC1_{i}-\sum^{\text{​}}PC1_{j},$$

where i is the score of signature for clusters with positive Cox coefficients and j is the expression of genes with negative Cox coefficients.

### GSEA and GSVA

We utilized gene set variation analysis (GSVA) (Hänzelmann et al., 2013) with the “GSVA” R package to explore different biological pathways in different metabolic clusters and different metabolic scores. We downloaded the gene sets of “c2.cp.kegg.v7.1.symbols.gmt” from the MSigDB database for conducting GSVA. Adjusted *p*-values less than 0.05 and |log2FC|>0.2 were considered statistically significant. Similarly, gene set enrichment analysis (GSEA) is a computational method for determining whether a largely defined set of genomes exhibits a statistically significant difference in the biological state (Subramanian et al., 2005). Based on the median expression value of prognostic metabolic signature genes (MSGs) in HP+ GC, the patients were classified into two subgroups, and the “c2.cp.kegg.v7.1.symbols.gmt” genomic enrichment analysis was performed, with a *p*-value < 0.05 regarded as statistically significant.

### Prediction of Drug Sensitivity

The chemotherapy drug sensitivity was accessed through the Genomics of Drug Sensitivity in Cancer (GDSC; https://www.cancerrxgene.org/) database (Yang et al., 2013). In addition, the half maximal inhibitory concentration (IC50) was estimated with the R package “pRRophetic” (Geeleher et al., 2014). In addition, we systematically searched for gene expression profiles for ICI treatment that were publicly available and combined with response outcome information. In our study, two cohorts of immunotherapy were eventually included: metastatic GC treated with pembrolizumab (an anti-PD-1 mcAb) (Kim S. T. et al., 2018) and melanoma treatment with ipilimumab (an anti-CTLA-4 mcAb) (Nathanson et al., 2017). Normalized gene expression profiles and response results of pretreatment biopsy samples were downloaded from Tumor Immune Dysfunction and Exclusion (TIDE, http://tide.dfci.harvard.edu./download/) for further analysis.

### Cell Culture and HP Infection

Human GC cell lines MKN45 were acquired from the Cell Resource Center, Institute of Biochemistry, and Cell Biology at the Chinese Academy of Science (Shanghai, China). MKN45 cells were cultured in RPMI-1640 (MACGENE, Beijing, China) containing 10% fetal bovine serum (Gibco, Carlsbad, CA, United States). All cultures were kept in a 5% CO_2_ humidified incubator at 37°C. HP strains 26695 and SS1 were kindly offered by Dr. Jihui Jia (Department of Microbiology, School of Basic Medical Science, Shandong University, Jinan, China). The HP strains were inoculated into Brucella broth supplemented with 5% FBS under microaerophilic conditions (5% O_2_, 10% CO_2_, and 85% N_2_) at 37°C. The bacteria were harvested and immediately transferred to cell cultures at a multiplicity of infection (MOI) of 100 and collected after 6 h.

### qRT-PCR Analysis

We extracted total RNA from the cells using RNA TRIzol reagent (CWBIO) to assess the expression levels of the five prognostic MRGs. cDNA synthesis was performed using a reverse transcription kit (CWBIO) according to the manufacturer’s instructions. Quantitative real-time polymerase chain reaction (qRT-PCR) analysis was conducted on a LightCycler 480 Real-Time PCR system. The relative mRNA expression level was calculated by using the 2^−ΔΔCt^ approach and standardized to β-actin. Supplementary Table S2 showed the primer sequences.

### Metabolomics Sample Collection and Preparation

A total of 37 serum samples were gathered from September 2019 to October 2020, provided by Jinan Central Hospital, all from fasted subjects. The study was approved by the Ethics Committee of the Jinan Central Hospital (Jinan, China) and performed in accordance with the relevant guidelines and regulations. After collection, all serum samples were kept at −80°C until use.

The study included the HP+ GC group (6 patients), HP GC group (19 patients), and HP− non-atrophic gastritis (NAG) group (12 patients). Clinical information of included patients was presented in Supplementary Table S3. The inclusion criteria for patients are given as follows: patients newly diagnosed with GC or NAG and had not received previous surgery, radiotherapy, or chemotherapy. The cancer or gastritis diagnosis was conducted by using the histopathological analysis of tissue specimens.

### LC–MS/MS Analysis and Data Processing

LC–MS/MS analyses were conducted by a UHPLC system (Vanquish, Thermo Fisher Scientific) with a UPLC BEH Amide column (2.1 mm × 100 mm, 1.7 μm) coupled to a Q Exactive HFX mass spectrometer (Orbitrap MS, Thermo). The mobile phase consisted of 25 mmol/L ammonium acetate and 25 mmol/L ammonia hydroxide in water (pH = 9.75) (A) and acetonitrile (B). The autosampler temperature was 4°C, and the injection volume was 3 μl.

A QE HFX mass spectrometer was used for its ability to acquire MS/MS spectra on the information-dependent acquisition (IDA) mode using the control of acquisition software (Xcalibur, Thermo). In this mode, acquisition software continuously evaluates the full scan MS spectrum. The ESI source conditions were set as follows: a sheath gas flow rate of 30 Arb, Aux gas flow rate of 25 Arb, capillary temperature of 350°C, full MS resolution of 60000, MS/MS resolution of 7500, collision energy of 10/30/60 in NCE mode, and spray voltage of 3.6 kV (positive) or −3.2 kV (negative).

The raw data were converted to the mzXML format using ProteoWizard and processed with an in-house program, which was developed using R and based on XCMS, for peak detection, extraction, alignment, and integration. Then an in-house MS2 database (BiotreeDB) was applied for metabolite annotation. The cutoff for annotation was set at 0.3.

To provide comparative interpretations and visualization of the metabolic differences between HP+ patients and HP− controls, principal component analysis (PCA) and orthogonal signal correction partial least-squares discriminant analysis (OPLS-DA) were applied. The quality of the model was described by R^2^X and Q^2^ values. Metabolites driving the difference in the metabolic profiles between the HP+ group and the HP− group were identified according to the variable importance in the projection (VIP) threshold of 1 from the OPLS-DA model.

### Statistical Analysis

Statistical analyses were performed using R language (version 4.0.4) or GraphPad Prism (version 6). Differences between the two groups were analyzed by using the Wilcoxon rank-sum test or Kruskal–Wallis rank-sum test. Data from qRT-PCR groups were described as mean (±) SD and evaluated by using two-tailed Student’s t-tests. The Kaplan–Meier method was adopted to generate survival curves for the subgroups in each dataset, and the log-rank (Mantel–Cox) test was applied to determine the statistical significance of the differences. We generated survival curves using the “survival” R package. Univariate analysis was performed using R depending on a Cox proportional hazard model *via* the survival package. Unless otherwise stated, the *p* values were two-sided, and *p* < 0.05 was considered statistically significant.

## Results

### Identification of Metabolic Pathway–Specific Genes in HP+ GC

**FIGURE 1:**
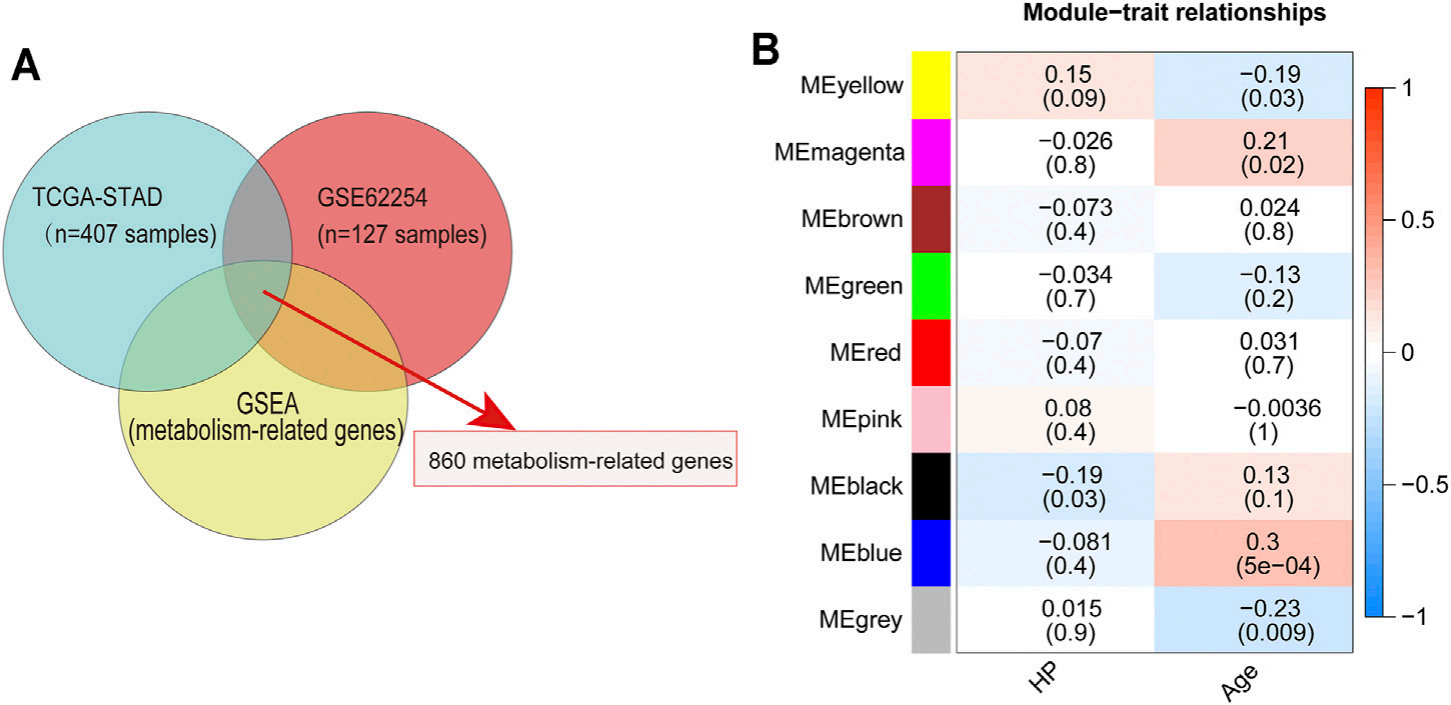
Identification of metabolism-related genes and WGCNA. **(A)** Venn diagrams of metabolism-related genes within the TCGA dataset, GEO dataset, and GSEA database. **(B)** Module–trait relationship heatmap based on the Pearson correlation coefficient between module eigengenes and clinical parameters (HP and age).

To identify MRGs that play an important role in the development of HP-induced GC, we first extracted the major metabolic pathway genes in GSEA using the TCGA and GEO databases to identify the intersections (Figure 1A). Subsequently, 860 MRGs (Supplementary Table S1) were selected for further analysis. Next, 127 GC samples containing HP infection information in GSE62254 were included in the WGCNA. In this study, a power of β = 4 (scale-free R^2^ = 0.87) was selected as the soft threshold (Supplementary Figure S1A). Then the hierarchical clustering tree for 860 MRGs was determined by conducting hierarchical clustering for dissTOM (Supplementary Figure S1B), and we identified the most significant correlation modules with clinical features. Finally, a total of nine modules were identified (Figure 1B). The black module was found to have the highest correlation with HP infection (Figure 1B), and these module genes (HP-MRGs, Supplementary Table S1) were selected for further analysis.

### Disease Connectivity Analysis and Metabolic Patterns Mediated by HP-MRGs

**FIGURE 2:**
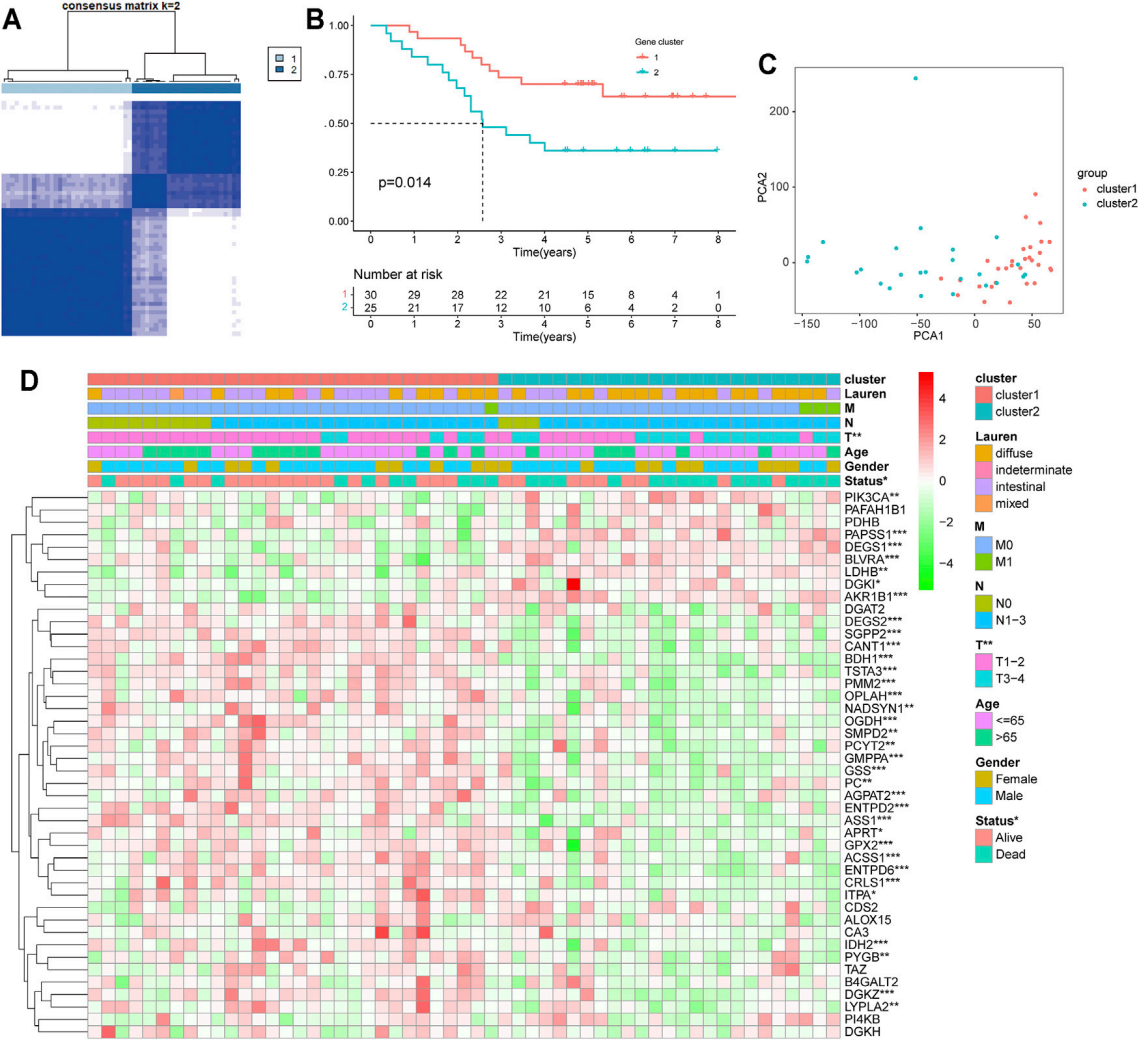
Differential clinicopathological features and overall survival of HP*-*induced gastric cancer in the Cluster 1/2 subgroups. **(A)** Consensus clustering matrix for k = 2. **(B)** Kaplan–Meier overall survival (OS) curves for patients in the Cluster 1/2 subgroup. **(C)** PCA plots for validation of the stability and reliability of the classification. **(D)** Heatmap and clinicopathologic features of the two clusters (Clusters 1/2) defined by the MRG consensus expression. *p < 0.05, **p < 0.01, ***p < 0.001.

Disease connectivity analysis showed that HP infection was significantly associated with metabolic disease, digestive system disease, cancer, and other diseases (Table 1), which demonstrates that HP may contribute to the development of disease by regulating the expression of HP-MRGs. To further explore the clinical relevance of 44 HP-MRGs in the black module, we clustered the HP-induced GC samples into subgroups based on the metabolic gene expression using the “ConsensusClusterPlus” R package. k = 2 was determined to have optimal clustering stability from k = 2 to 9 based on the expression similarity of 44 HP-MRGs and the proportion of ambiguous clustering measures (Figure 2A; Supplementary Figures S1C,D, S4). Accordingly, a total of 55 HP+ GC samples were clustered into two subtypes (Cluster 1: *n* = 30 and Cluster 2: *n* = 25). The overall survival (OS, *p* = 0.014) of Cluster 1 was longer than that of Cluster 2 (Figure 2B), indicating that these HP-MRGs could classify the HP+ GC samples at a prognostic level. Similarly, HP− GC samples of GEO and TCGA database were also performed based on 44 HP-MRGs using the “ConsensusClusterPlus” R package (Supplementary Figures S2–S6). And HP− GC samples were divided into two subtypes (Cluster 1 and Cluster 2), respectively. However, there was no significant prognostic difference between Cluster 1 and Cluster 2 in the HP− GC samples (Supplementary Figures S2D, S3D). For clarifying the mechanism of the subgroup difference in HP+ and HP− samples, we further performed gene ontology biological processes and pathway enrichment analysis of 44 HP-MRGs in the black module *via* Metascape (http://metascape.org/), which is an effective and efficient tool for experimental biologists to comprehensively analyze (Zhou et al., 2019). Not surprisingly, we found that most HP-MRGs were enriched in the metabolic process (Supplementary Figures S1E,F). Meanwhile, response to a stimulus, response to acid chemical, and neutrophil degranulation were also enriched (Supplementary Figures S1E,F), consistent to the results of WGCNA analysis. These findings suggested that HP-MRG is involved in both metabolic processes and processes in response to external stimuli and inflammatory responses, which reveals why the HP+ samples get such clinical signature. Moreover, the gene expression pattern was analyzed between the two subtypes by using the principal component analysis (PCA) method (Figure 2C), and we found that the gene expression profiles between the two subtypes were differentiated well. In addition, the clinicopathological features between the two subtypes were also compared (Figure 2D). As shown in the heatmap, the expression of most HP-associated MRGs differed significantly between the two subtype groups. Cluster 2 was preferentially associated with a high TNM-T stage (*p* < 0.01). Multivariable cox regression analysis revealed that the TNM stage is an important factor affecting prognosis of HP infection–induced GC patients (Supplementary Table S4). The previous findings revealed that the clustering metabolic subtypes defined by HP-MRGs were closely related to the heterogeneity of GC patients with HP infection.

**TABLE 1:** Top 24 enriched diseases of 44 HP-MRGs analyzed with the Comparative Toxicogenomics Database.

Disease name	Disease categories	*p*-value	Corrected *p*-value	Annotated genes quantity	Annotated genes	Genome frequency
Metabolism, inborn errors	Genetic disease (inborn)|metabolic disease	3.37E-12	1.09E-09	12	AGPAT2|APRT|ASS1|GSS|IDH2|ITPA|LDHB|OGDH|OPLAH|PC|PDHB|PMM2	706/44146 genes: 1.60%
Nutritional and metabolic diseases		3.17E-11	1.02E-08	15	AGPAT2|APRT|ASS1|CA3|ENTPD6|GSS|IDH2|ITPA|LDHB|OGDH|OPLAH|PC|PDHB|PIK3CA|PMM2	1656/44146 genes: 3.75%
Pathological conditions, signs, and symptoms		1.4E-09	4.53E-07	18	AKR1B1|ALOX15|BDH1|BLVRA|CA3|CANT1|DGKZ|ENTPD2|ENTPD6|GMPPA|GPX2|IDH2|ITPA|LDHB|PAFAH1B1|PC|PIK3CA|PMM2	3421/44146 genes: 7.75%
Metabolic diseases	Metabolic disease	2.04E-09	6.59E-07	13	AGPAT2|APRT|ASS1|GSS|IDH2|ITPA|LDHB|OGDH|OPLAH|PC|PDHB|PIK3CA|PMM2	1540/44146 genes: 3.49%
Genetic diseases, inborn	Genetic disease (inborn)	2.55E-09	8.22E-07	15	AGPAT2|APRT|ASS1|CANT1|GMPPA|GSS|IDH2|ITPA|LDHB|OGDH|OPLAH|PC|PDHB|PIK3CA|PMM2	2275/44146 genes: 5.15%
Congenital, hereditary, and neonatal diseases and abnormalities		8.69E-09	2.81E-06	16	AGPAT2|APRT|ASS1|CANT1|GMPPA|GSS|IDH2|ITPA|LDHB|OGDH|OPLAH|PAFAH1B1|PC|PDHB|PIK3CA|PMM2	2912/44146 genes: 6.60%
Pathologic processes	Pathology (process)	3.42E-08	1.11E-05	14	AKR1B1|BDH1|BLVRA|CA3|CANT1|DGKZ|ENTPD2|GPX2|IDH2|ITPA|LDHB|PAFAH1B1|PC|PIK3CA	2342/44146 genes: 5.31%
Digestive system diseases	Digestive system disease	3.99E-08	1.29E-05	15	AKR1B1|ALOX15|ASS1|BDH1|BLVRA|CA3|DGAT2|ENTPD2|GMPPA|GPX2|LDHB|OGDH|PC|PIK3CA|PMM2	2793/44146 genes: 6.33%
Liver diseases	Digestive system disease	3.25E-07	0.000105	12	AKR1B1|ASS1|BDH1|BLVRA|CA3|DGAT2|ENTPD2|GPX2|LDHB|OGDH|PC|PIK3CA	1964/44146 genes: 4.45%
Neoplasms	Cancer	1.7E-06	0.00055	15	AKR1B1|ALOX15|APRT|DEGS1|ENTPD6|GPX2|GSS|IDH2|LDHB|PAFAH1B1|PC|PDHB|PIK3CA|PMM2|PYGB	3736/44146 genes: 8.46%
Carcinoma, renal cell	Cancer|urogenital disease (female)|urogenital disease (male)	9.17E-06	0.00296	4	APRT|LDHB|PDHB|PIK3CA	131/44146 genes: 0.30%
Amino acid metabolism, inborn errors	Genetic disease (inborn)|metabolic disease	1.33E-05	0.0043	4	ASS1|GSS|OGDH|OPLAH	144/44146 genes: 0.33%
Liver cirrhosis	Digestive system disease|pathology (process)	2.74E-05	0.00885	7	AKR1B1|BDH1|BLVRA|CA3|ENTPD2|LDHB|PC	895/44146 genes: 2.03%
Kidney neoplasms	Cancer|urogenital disease (female)|urogenital disease (male)	4.11E-05	0.01327	4	APRT|LDHB|PDHB|PIK3CA	192/44146 genes: 0.43%
Fibrosis	Pathology (process)	4.19E-05	0.01354	7	AKR1B1|BDH1|BLVRA|CA3|ENTPD2|LDHB|PC	957/44146 genes: 2.17%
Brain diseases, metabolic, inborn	Genetic disease (inborn)|metabolic disease|nervous system disease	0.000052	0.0168	4	ASS1|IDH2|PC|PDHB	204/44146 genes: 0.46%
Chemical and drug-induced liver injury	Digestive system disease	5.44E-05	0.01756	5	BDH1|CA3|DGAT2|OGDH|PC	410/44146 genes: 0.93%
Kidney diseases	Urogenital disease (female)|urogenital disease (male)	5.98E-05	0.01931	6	APRT|DGKH|IDH2|LDHB|PDHB|PIK3CA	688/44146 genes: 1.56%
Brain diseases, metabolic	Metabolic disease|nervous system disease	9.14E-05	0.02951	4	ASS1|IDH2|PC|PDHB	236/44146 genes: 0.53%
Diabetes complications	Endocrine system disease	0.000109	0.03522	3	AKR1B1|ASS1|DGKH	92/44146 genes: 0.21%
Pyruvate metabolism, inborn errors	Genetic disease (inborn)|metabolic disease	0.000115	0.0373	2	PC|PDHB	16/44146 genes: 0.04%
Liver cirrhosis, experimental	Digestive system disease|pathology (process)	0.000116	0.03761	6	AKR1B1|BDH1|BLVRA|CA3|LDHB|PC	777/44146 genes: 1.76%
Neoplasms by site	Cancer	0.000117	0.03763	11	AKR1B1|ALOX15|APRT|DEGS1|GPX2|GSS|LDHB|PDHB|PIK3CA|PMM2|PYGB	2958/44146 genes: 6.70%
Neoplastic processes	Cancer|pathology (process)	0.000133	0.04289	5	GPX2|IDH2|LDHB|PAFAH1B1|PIK3CA	496/44146 genes: 1.12%

HP-MRGs: *Helicobacter pylori*–associated metabolism-related genes.

### Metabolic Patterns Characterized by Distinct Immune Landscapes

**FIGURE 3:**
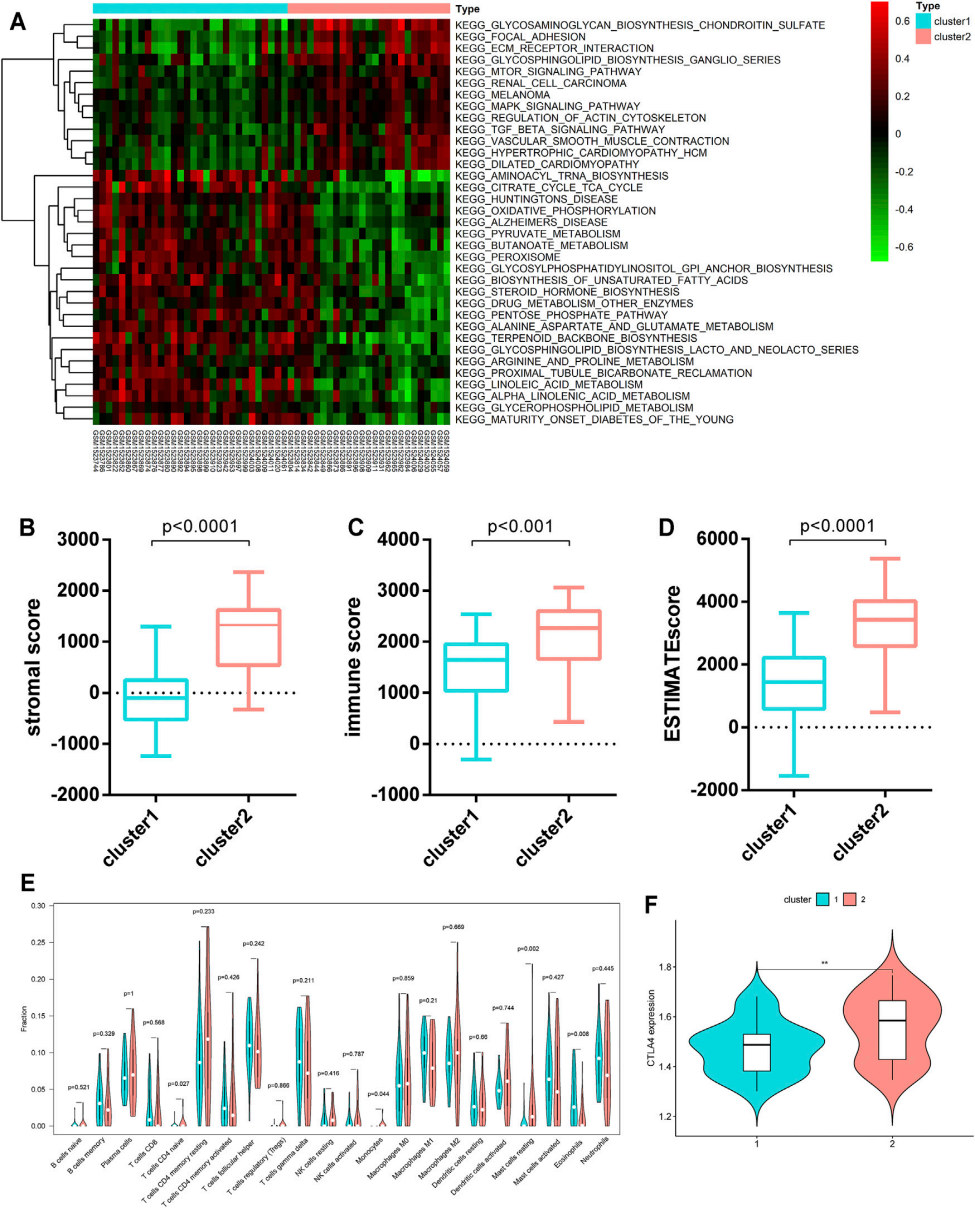
TME characteristics and relevant biological pathways in Cluster 1/2 subtypes. **(A)** GSVA enrichment analysis showing the activation states of biological pathways in the Cluster 1/2 subtypes. **(B–D)** The stromal score, immune score, and ESTIMATE score of the two clusters were analyzed and plotted. **(E)** The infiltrating levels of 22 immune cell types in Cluster 1/2 subtypes (assume blue is Cluster 1 and red is Cluster 2). The significant differences of the three gene clusters were compared through the Kruskal–Wallis H test. **(F)** Comparison of the CTLA4 expression levels across the two clusters.

To discover the biological behaviors among these distinct metabolic patterns (Clusters 1/2), we carried out GSVA enrichment analysis. As shown in Figure 3A, Cluster 2 was markedly enriched in carcinogenic and stromal activation pathways such as the TGF-β signaling pathway, ECM receptor interaction, focal adhesion, mTOR signaling pathway, and MAPK signaling pathways. However, Cluster 1 presented enrichment pathways prominently associated with metabolic pathway activation, such as the citrate cycle (TCA cycle), pyruvate metabolism, and peroxisomes. Several previous studies have reported that such immune rejection tumors are characterized by an abundant population of immune cells that are preserved in the stroma surrounding the nests of tumor cells, rather than penetrating their parenchyma (Chen and Mellman, 2017). Furthermore, the ESTIMATE algorithm was applied to quantify the overall infiltration of immune cells (immune score), stromal cells (stromal score), and tumor cell purity (ESTIMATE score) in two clusters. Intriguingly, the results showed that Cluster 2 exhibited significantly higher stromal scores, immune scores, and ESTIMATE scores than Cluster 1 (Figures 3B–D), suggesting that Cluster 2 was surrounded by more non-tumor components (e.g., immune cells and stromal cells).

We further evaluated the immune infiltration profile by CIBERSORT, a deconvolution algorithm for assessing the immune cell landscape in the tumor microenvironment using support vector regression. A consistent result was also observed in the expression stratification of such HP-MRGs. Monocytes and resting mast cells were significantly more enriched in Cluster 2 than in Cluster 1 (Figure 3E). Taken together, the aforementioned analysis demonstrated that Cluster 2 was classified as an immune-excluded phenotype featuring reduced immune cell infiltration and stromal activation. Cluster 1 is an immune-desert phenotype characterized by immune suppression and activation of metabolic pathways associated with immune regulation. Considering that CTLA-4 is a well-documented indicator to predict the treatment response to anti-CTLA-4, we also compared the CTLA-4 expression level between the two clusters and observed a significant upregulation of the CTLA-4 expression in Cluster 2. We were surprised to confirm that the different metabolic patterns were also characterized by different immune infiltration profiles.

### Metabolic Score Construction and Its Clinical Relevance

**FIGURE 4:**
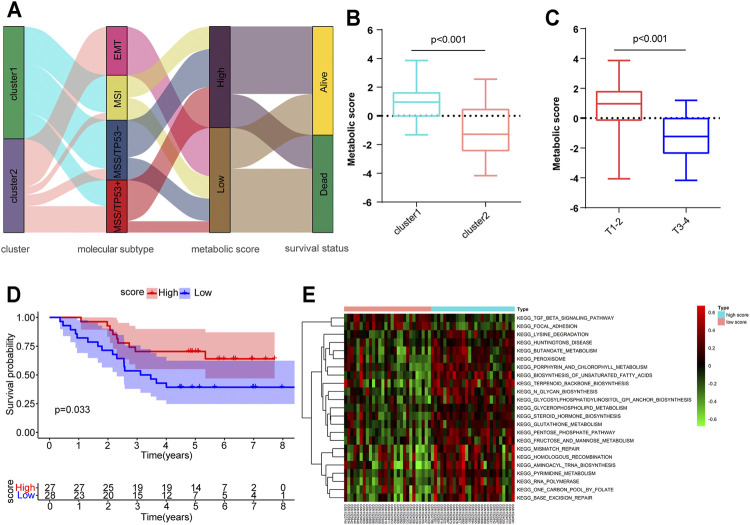
Construction of the metabolic score and exploration of the relevance of clinical features and biological pathways. **(A)** Alluvial diagram of metabolism-related gene clusters in groups with different ACRG subtypes (EMT, MSI, MSS/TP53^-^, and MSS/TP53^+^), metabolic scores, and survival outcomes. **(B,C)** Metabolic score differences in the Cluster 1/2 subtypes and different tumor T stages. **(D)** Kaplan–Meier curves for high (*n* = 27) and low (*n* = 28) metabolic score groups of HP*-*induced gastric cancer. **(E)** GSVA enrichment analysis showing the activation states of biological pathways in high– and low–metabolic score subtypes.

Although we have determined the role of distinct metabolic patterns in prognosis and immune infiltration based on HP-MRG expression, these analyses are population-based and do not precisely predict the metabolic patterns of personalized tumors. Hence, we established a scoring method named the metabolic score, derived from the identified MSGs (Supplementary Table S1), to quantify the metabolic pattern of individual HP-induced GC. An alluvial diagram was applied to visualize the attribute changes of individual patients (Figure 4A). These results indicated that patients with EMT subtypes, all in Cluster 2, had the lowest metabolic score compared to the other three molecular subtypes. Comparing the performance of cluster and molecular subtypes in terms of the gene expression, we found a partial consistency in the expression of key genes between molecular subtypes and clusters, especially EMT subtypes and Cluster 2 (Supplementary Figure S8A; Supplementary Table S5). Next, the alluvial diagram was applied to visualize the attribute changes of individual patients in HP− GC samples of GEO and TCGA database (Supplementary Figures S8B,C). The results showed that the large variability in the distribution of ACRG subtypes between HP+ and HP− samples of the GEO database, suggesting that the subtypes are more specific in HP+ samples. Notably, Cluster 1 showed a higher metabolic score than Cluster 2 (Figure 4B), as exhibited by processes related to the activation of many metabolic pathways in Cluster 1 (Figure 3A), further corroborating the correctness of the scoring scheme. We also classified HP+ GC based on MSI and MSS provided by GSE62254. As expected, Cluster 2 has a higher percentage of MSS than Cluster 1 (Supplementary Figure S7), which suggest that such genetic alternation between clusters may be the main “driver” for OS difference. Subsequently, patients with a high TNM-T stage were linked to a low metabolic score (Figure 4C), consistent with the previous result that metabolic patterns were related to the TNM-T stage. As anticipated, patients with high metabolic scores were significantly correlated with a more favorable prognosis (*p* = 0.033, Figure 4D). However, the metabolic score was not an independent factor for OS in HP+ GC (Supplementary Table S4). Similarly, we conducted the GSVA enrichment analysis to explore the biological behaviors between high and lowmetabolic scores. As shown in the heatmap, a high metabolic score was significantly correlated with metabolic pathway activation, such as the glutathione metabolism and fructose and mannose metabolism (Figure 4E). In short, the metabolic score allows for a better assessment of the metabolic pattern of individual tumors.

### Expression, Prognosis, and GSEA of MSGs in HP+ GC

**FIGURE 5:**
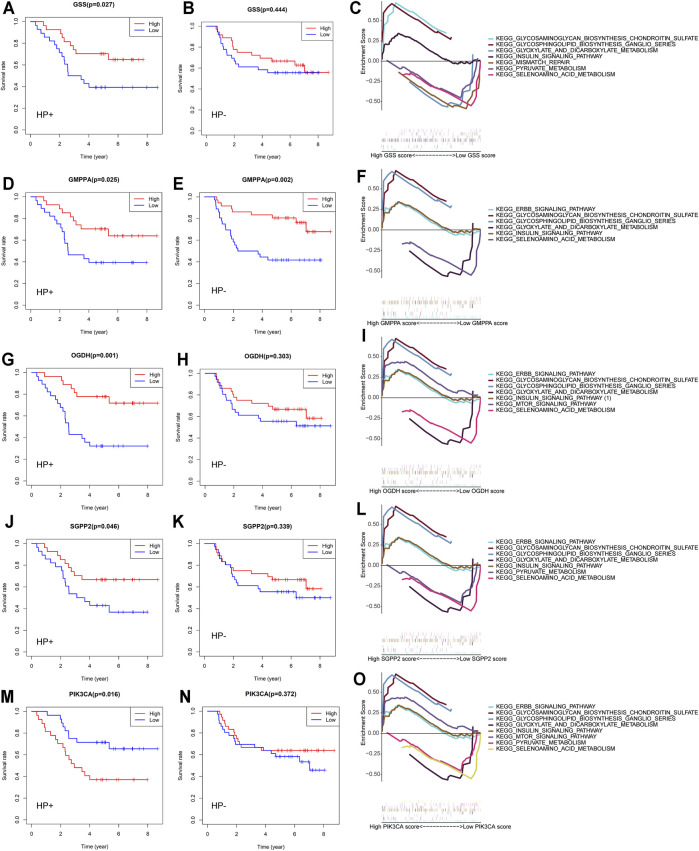
Overall survival and GSEA of five MSGs in GC patients. Overall survival outcomes in HP+ and HP− GC patients dichotomized by median **(A,B)** GSS expression, **(D,E)** GMPPA expression, **(G,H)** OGDH expression, **(J,K)** SGPP2 expression, and (M, N) PIK3CA expression. Enrichment plots from the gene set enrichment analysis (GSEA) between high and low **(C)** GSS expression, **(F)** GMPPA expression, **(I)** OGDH expression, **(L)** SGPP2 expression, and **(O)** PIK3C expression groups in HP+ GC patients. MSGs: metabolic signature genes.

Tumor-related alterations in metabolism have functional consequences on the progression of disease, treatment effects, and survival outcomes (Vander Heiden and DeBerardinis, 2017). We dichotomized the expression profiles for each of the 25 previously selected MSGs by the median expression and calculated the effect of high vs low expression on the OS of HP+ GC patients. We confirmed five genes that were significantly related to patient survival in HP+ GC patients (Figures 5A,D,G,J,M). These genes were GSS, which encodes the proteins that function as a homodimer to catalyze the second step of glutathione biosynthesis; GMPPA, which encodes a GDP–mannose pyrophosphorylase; OGDH, which encodes one subunit of the 2-oxoglutarate dehydrogenase complex located in the mitochondrial matrix; SGPP2, which encodes a transmembrane protein that degrades the bioactive signaling molecule sphingosine-1-phosphate (S1P) and is induced during inflammatory responses; and PIK3CA, an integral part of the PI3K pathway. Thus, detailed studies of GSEA in HP+ GC are presented.

Previous studies indicated that HP infection could increase the production of reactive oxygen species (ROS) and decrease the levels of glutathione (GSH) in gastric epithelial cells (Wada et al., 2018). The heightened expression of GSS, involved in the glutathione metabolism, is correlated with increased HP+ GC patient survival (Figure 5A) but not HP-negative (HP−) GC (Figure 5B). GSEA revealed that the differential expression of GSS in HP+ GC was associated with glyoxylate and dicarboxylate metabolism, pyruvate metabolism, and selenoamino acid metabolism (Figure 5C).

Similar to GSS, the elevated expression of GMPPA, OGDH, and SGPP2, which plays important roles in the energy metabolism and the inflammatory response, was also correlated with better HP+ GC patient survival (Figures 5D,G,J). However, the OGDH and SGPP2 expression in HP− GC was not significantly associated with patient survival (Figures 5E,H,K). Likewise, GSEA suggested that the differential expression of these three genes in HP+ GC was coenriched in key metabolism-related pathways, such as glyoxylate and dicarboxylate metabolism, pyruvate metabolism, and selenoamino acid metabolism (Figures 5F,I,L).

In contrast to GSS, GMPPA, OGDH, and SGPP2, low expression of PIK3CA was associated with better OS outcomes in patients with HP+ GC (Figure 5M) but not in patients with HP− GC (Figure 5N). Similarly, GSEA showed that the differential expression of PIK3CA in HP+ GC was enriched in metabolism-related pathways, such as glyoxylate and dicarboxylate metabolism, pyruvate metabolism, and mTOR signaling pathway (Figure 5).

**FIGURE 6:**
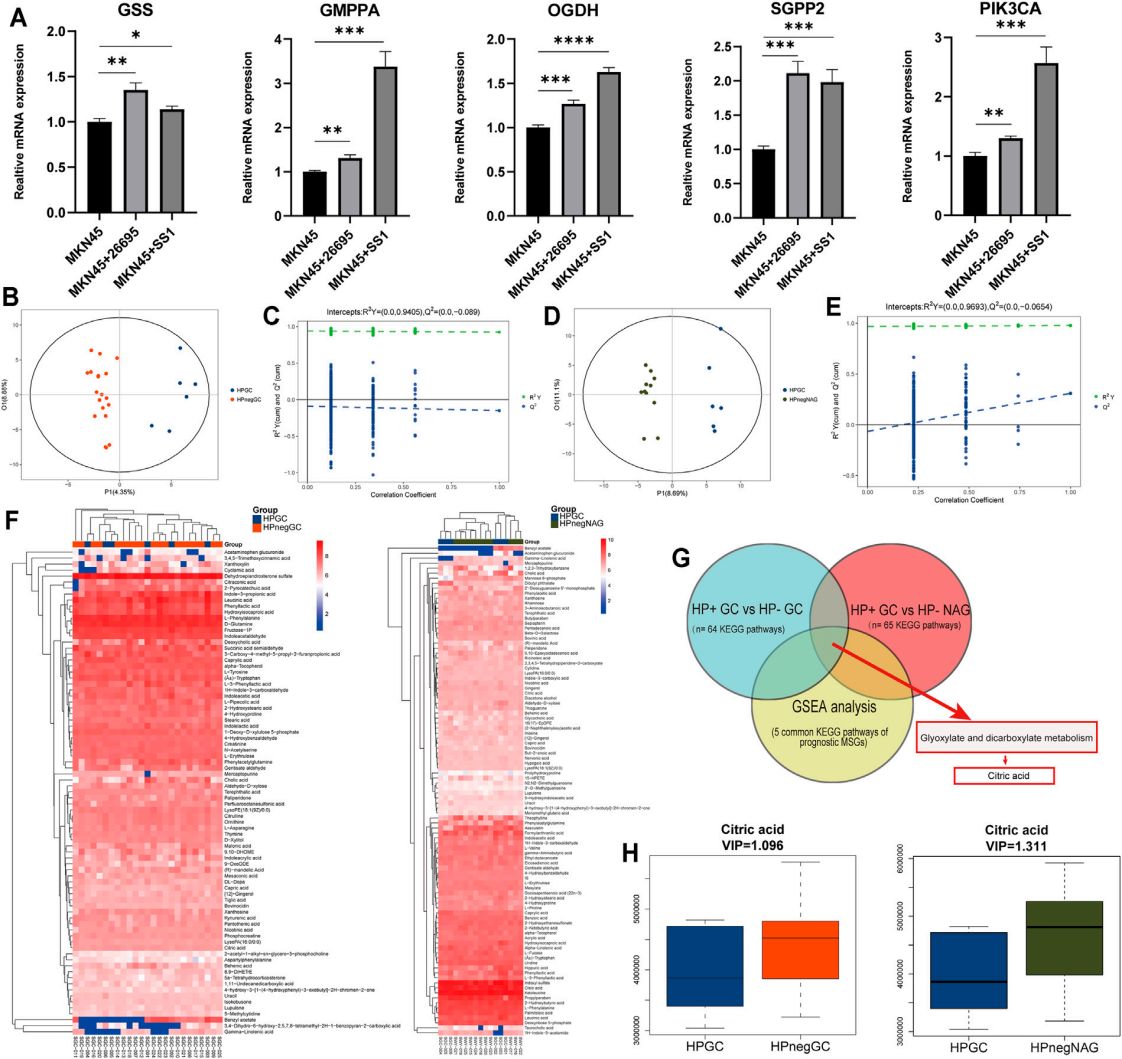
PCR analysis and identification of differential metabolites and pathways *via* prognostic MSGs induced by HP. **(A)** qRT-PCR analysis of the mRNA levels of the five MRGs in MKN45 cells. **(B, D)** Score plots for the first three latent components of the PLS-DA model for HP GC vs. HP neg GC **(B)** and HP GC vs. HP neg NAG **(D)**. **(C, E)** Results of the 1,000 times permutation test of the OPLS-DA model for HP GC vs. HP neg GC **(B)** and HP GC vs. HP neg NAG **(D)**. **(F)** Heatmap for relative abundances of all identified differential metabolites in the HP GC vs. HP neg GC and HP GC vs. HP neg NAG. **(G)** Venn diagrams of KEGG pathways within HP GC vs. HP neg GC, HP GC vs. HP neg NAG and GSEA. **(H)** Boxplot depicting the expression level of citric acid in the HP GC vs. HP neg GC and HP GC vs. HP neg NAG subgroups. *p < 0.05, **p < 0.01, ***p < 0.001, ***p < 0.0001 by Student’s t-test. HP GC: *Helicobacter pylori*–positive gastric cancer; HP neg GC: *Helicobacter pylori*–negative gastric cancer; HP neg NAG: *Helicobacter pylori*–negative non-atrophic gastritis.

To further ascertain the extent to which each of the HP+ GC survival–related MRGs could influence patient outcomes, we generated a hazard ratio (HR) for each gene and the various clinical variables by univariate and multivariate analyses (Table 2). As expected, the HRs of GSS, GMPPA, OGDH, and SGPP2 indicated a reduced risk of death, while the opposite was true for PIK3CA. However, only the TNM stage is an independent prognostic factor in HP+ GC patients. Meanwhile, in MKN45 cells, HP infection markedly upregulated the expression of the five MRGs (Figure 6A). Taken together, these five genes are closely associated with HP infection and patient survival.

**TABLE 2:** Univariate and multivariate analyses of the correlation of clinical variables and expression of metabolic genes with overall survival in HP+ GC.

Parameter	Variables	Univariate analysis		Multivariate analysis	
		HR (95% CI)	*p* value	HR (95% CI)	*p* value
Sex	Male vs. Female	1.153 (0.514–2.589)	0.73	2.12 (0.742–6.076)	0.16
Age	≤65 vs. > 65 (years old)	0.541 (0.227–1.291)	0.166	0.83 (0.303–2.281)	0.72
T stage	T1-2 vs. T3-4	3.37 (1.549–7.335)	0.002	3.84 (1.308–11.301)	0.01
N stage	N0 vs. N1-3	2.651 (0.794–8.850)	0.113	2.84 (0.681–11.863)	0.15
M stage	M0 vs. M1	8.637 (2.744–27.189)	<0.001	11.10 (2.252–54.672)	0.003
PIK3CA	Expression (low vs. high)	2.611 (1.161–5.873)	0.02	2.84 (0.941–8.588)	0.06
OGDH	Expression (low vs. high)	0.25 (0.104–0.599)	0.002	0.83 (0.275–2.554)	0.75
SGPP2	Expression (low vs. high)	0.449 (0.200–1.008)	0.052	1.11 (0.342–3.580)	0.87
GMPPA	Expression (low vs. high)	0.407 (0.181–0.918)	0.03	0.87 (0.315–2.425)	0.80
GSS	Expression (low vs. high)	0.412 (0.183–0.93)	0.032	0.83 (0.253–2.719)	0.76

### Identification of Differential Metabolites and Pathways *via* Prognostic MSGs Induced by HP

Metabolic profiling conducted in this research included sample preparation, metabolite extraction, and LC/MS analysis. Typical total ion current (TIC) chromatograms of quality control (QC) samples analyzed by UHPLC-QE-MS in the positive mode (ESI+) or negative mode (ESI-) are presented in Supplementary Figure S9A,B, respectively. In this study, original data in the ESI mode were chosen for further analysis. We first checked the experimental system by including the QC samples in PCA. As presented in Supplementary Figures S9C,D, all QCs are concentrated in the center of the axes, suggesting the stability of the analytical system and the reliability of the data. On the basis of the class information, PLS-DA with better discriminative power than PCA was performed to describe the metabolic profile. Thus, an OPLS-DA model was developed. Comparison of all pairs using the score plots of the first three potential components of the PLS-DA model showed significant clustering, which indicated a clear separation (Figures 6B,D). No overfitting for the negative mode was noticed according to the permutation validation (Figures 6C,E). These results confirmed the high goodness of fit and predictive capability of the PLS-DA models. Then a total of 81 differentially accumulated metabolites in the HP+ GC vs. HP− GC group and 98 differential metabolites in the HP+ GC vs. HP− NAG group were identified depending on the variable importance in the projection (VIP) > 1 in the loading plot. A general overview of the metabolic profile is shown in the heatmap, which includes 81 and 98 different metabolites in the two groups (Figure 6F). Next, we performed the KEGG pathway analysis of differential metabolites in the two groups. The results revealed that 64 KEGG pathways and 65 KEGG pathways were enriched (Figure 6G). To determine which metabolites or pathways HP infection acts on by affecting prognostic MSGs, we took intersections of the enriched pathways and GSEA. Interestingly, the glyoxylate and dicarboxylate metabolism pathway, which is a carbohydrate metabolism pathway, was identified, and citric acid was a common differential metabolite in this pathway (Figure 6G). We found that the expression level of citric acid was decreased in both groups after HP infection (Figure 6H). Therefore, the aforementioned results suggested that the carbohydrate metabolism and citric acid may be downstream regulators of the function of metabolic genes in HP-induced GC. A previous study reported that citric acid showed a potent inhibitory activity on growth of *Helicobacter pylori* strains *in vitro*.

### Predictive Value of Prognostic MSGs as Biomarkers for Therapeutic Effects

**FIGURE 7:**
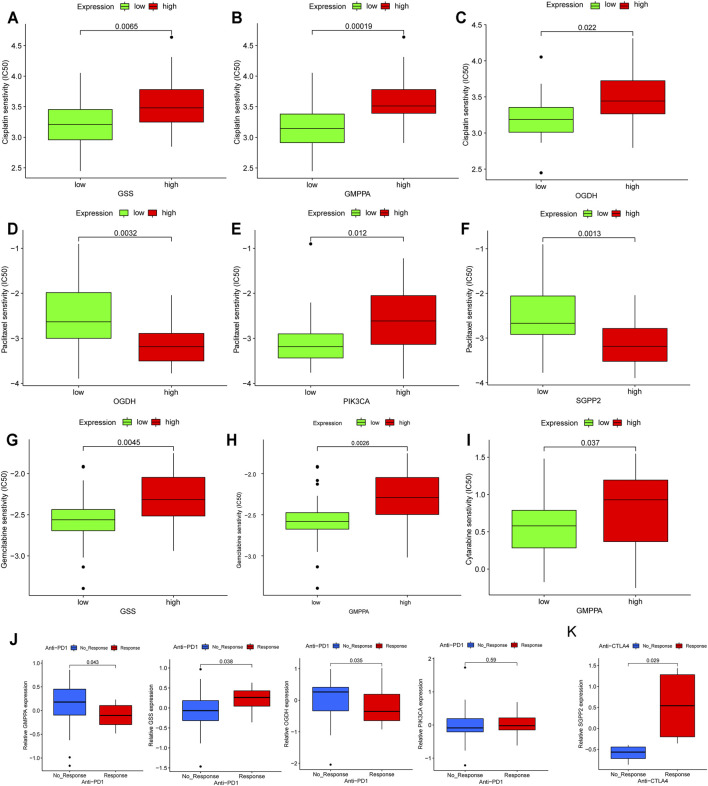
Assessing the immuno-/chemotherapeutic response of high and low expression of the five MSGs. **(A–C)** Box plots for the estimated IC50 of cisplatin in high and low **(A)** GSS, **(B)** GMPPA, and **(C)** OGDH expression. **(D–F)** Box plots for the estimated IC50 of paclitaxel in high and low **(D)** OGDH, (E) PIK3CA and **(C)** SGPP2 expression. **(G,H)** Box plots for the estimated IC50 of gemcitabine in high and low **(G)** GSS and (H) GMPPA expression. (I) Box plots for the estimated IC50 of cytarabine in high and low GMPPA expression. **(J)** The proportion of metastatic GC patients who responded to PD-1 blockade immunotherapy with low or high expression of GSS, GMPPA, OGDH, and PIK3CA. **(K)** The fraction of melanoma patients with a clinical response to anti-CTLA-4 immunotherapy in the low- or high-SGPP2 expression groups. MSGs: metabolic signature genes.

Based on the fact that these five genes (GSS, GMPPA, SGPP2, OGDH, and PIK3CA) have prognostic utility in HP+ GC, they are attractive therapeutic targets and deserve further exploration, especially considering that treatment with chemotherapy and immunotherapy drugs may phenocopy the effects of the expression of these metabolic genes. Thus, we first investigated conventional chemotherapy drug sensitivity in HP+ GC. In detail, the estimated IC50 levels of cisplatin were distinctly lower in low expression than in high expression of GSS, GMPPA, and OGDH, suggesting that these three genes could be used as biomarkers whose low expression indicates the tumor is more sensitive to cisplatin (Figures 7A–C). Similarly, we found that high expression of OGDH and SGPP2 was correlated with being more sensitive to paclitaxel, but the opposite was true for PIK3CA (Figures 7D–F).

Additionally, we estimated the IC50 levels of two antimetabolic chemotherapeutic agents, gemcitabine and cytarabine, in the high- and low-expression groups of five prognostic MSGs. The results showed that the low expression of GSS and GMPPA was correlated with higher sensitivity to gemcitabine (Figures 7G,H), while low expression of GMPPA indicated higher sensitivity to cytarabine (Figure 7I).

Currently, immunotherapy, such as immune checkpoint inhibitor therapy, is increasingly being used for cancer treatment, and it can be synergistic with chemotherapy (Yang, 2015). We first examined the distribution of prognostic MSG expression among five immune subtypes reported by a recent study (Thorsson et al., 2018) and found that there was a significant difference in GSS, GMPPA, SGPP2, and PIK3CA among the five immune subtypes (Supplementary Figure S10), suggesting that most prognostic MSGs are closely associated with the tumor immune microenvironment. Then in the anti-PD-1 cohort (Nathanson et al., 2017), patients with high expression of GSS and PIK3CA exhibited significant clinical advantages (Figure 7J). Efficacy and immune response to anti-PD-1 treatment were demonstrated in patients with low GMPPA and OGDH expression compared to those with high expression (Figure 7J). In the anti-CTLA-4 cohort (Kim S. T. et al., 2018), higher SGPP2 expression in melanoma patients was associated with a better therapeutic benefit (Figure 7K). Taken together, our findings strongly suggest that the expression of these five genes is associated with the response to immune/chemotherapy.

## Discussion

Increasing evidence has shown that HP infection significantly affects metabolic changes in the host (Niemelä et al., 1996; Buzás, 2014). Although studies have revealed the important role of MRGs and metabolism-based models for predicting GC survival outcomes (Luo et al., 2020; Wen et al., 2020), altered metabolic profiles in HP-induced GC have not been comprehensively recognized. The alterations of metabolism-related genes enable cancer cells to reprogram the metabolism to meet the increased energy demands for survival. Therefore, identifying HP infection–related metabolic signatures, which influence metabolic activities, signaling cascades, and tumor progression, will provide novel insights into HP-induced GC and delineate multiple effective strategies for therapeutic intervention.

Rapidly growing tumor cells require essential metabolites to proliferate and create an immunosuppressive microenvironment. How HP infection alters immunometabolism remains unclear. In the present study, based on the HP infection status, we identified two distinct metabolic patterns (Clusters 1/2) characterized by different immune phenotypes (immune desert and immune exclusion phenotypes), which were associated with diverse anticancer immunity. Of note, Cluster 2 was classified as an immune-excluded phenotype and had worse T staging and immune cell suppression status, leading to a worse prognosis. The immune-desert phenotypes were correlated with immune tolerance and minimal activation of cancer-specific T cells (Kim and Chen, 2016). Regarding the immune-excluded phenotype, the stroma may be confined within the tumor envelope or may penetrate the tumor itself, making it appear that the immune cells are actually inside the tumor (Gajewski, 2015; Joyce and Fearon, 2015). A recent report revealed that the TME plays an important role in the progression of tumors and immunotherapy responsiveness (Binnewies et al., 2018). As shown in our data, the Cluster 2 pattern was significantly related to increased levels of tumor-infiltrating monocytes and CTLA-4, supporting the potential predictive value of immunotherapy for HP-induced GC.

GC is a heterogeneous disease with distinct clinical behaviors and risk burdens. Genomic and transcriptomic analyses have identified heterogeneity within GC and classified it into molecular subtypes characterized by specific genetic aberrations and expression profiles indicating the presence of important biological differences (Cristescu et al., 2015). Therefore, we established a metabolic score to quantify the metabolic patterns of molecular subtypes for a better classification of prognostic prediction. A previous study reported that the diffuse histological type and EMT molecular subtype were markedly associated with worse survival, while MSI was associated with better clinical outcomes in gastric cancer (Zhang et al., 2020). Our study identified 25 potential “subtype biomarkers” or metabolic signature genes (MSGs) and established a metabolic score to quantify the metabolic pattern. Accordingly, the metabolic model featuring the immune-excluded phenotype displayed a lower metabolic score and more EMT subtypes, while the model featuring the immune-desert form exhibited a higher MSI subtype and higher metabolic score. Through GSVA, genes involved in immunosuppressive pathways, such as the TGF-β signaling pathway, were clearly enriched in the low–metabolic score group. The previous results suggested that HP infection may exacerbate the close association between the immune microenvironment and metabolic patterns. Therefore, HP+ GC patients with low metabolic scores have a worse prognosis. These findings may help improve our understanding of the mechanisms underlying the formation of different metabolic patterns in HP+ GC.

Recent research demonstrated that the alteration of specific transcripts of metabolic genes could act as a predictive biomarker of survival outcomes (Prusinkiewicz et al., 2020). Similarly, our analysis found that the expression of five MRGs (GSS, GMPPA, OGDH, SGPP2, and PIK3CA) was predictive of survival for HP+ GC. We further confirmed that HP infection remarkably upregulated the expression of five MRGs in MKN45 GC cells by using two HP strains. Our data are consistent with other emerging evidence showing that the intracellular glutathione (GSH) level was decreased and the GSS mRNA expression was downregulated in HP-infected AGS GC cells (Matsuoka et al., 2020). Our findings further confirmed that the GSS expression may be important for survival outcomes only in HP+ GC.

GMPPA, which is involved in glycolytic processes, has been implicated as a negative prognostic factor in several tumor types (Cho et al., 2018; Zhao et al., 2021). However, this is the first time that GMPPA has been found to play an important role in HP-induced GC.

OGDH is the first rate-limiting E1 subunit of the OGDH complex (OGDHC), which serves as a regulatory point in the crossroad of the TCA cycle and glutamine metabolism. The host produces effector molecules such as reactive oxygen species (ROS) to counteract HP infection. Interestingly, OGDH played a crucial role in the interdependent homeostasis of ROS and NADPH in GC cells (Lu et al., 2019). We speculated that OGDH might be the regulatory site of ROS destabilization in response to HP infection.

SGPP2, one isoform of S1P phosphatases, was reported to be remarkably associated with intestinal epithelial integrity and bacterial infiltration into the mucosa (Huang et al., 2016). To date, little is known about the function of SGPP2 in HP-induced GC.

PIK3CA, a member of the phosphatidylinositol 3’-kinase (PI3K) family, is ranked as the second most commonly mutated oncogene, detected in more than 10% of patients with eight types of cancer (Arafeh and Samuels, 2019). By GSEA, we found that high expression of the five prognostic MRGs was more enriched in cancer and bacteria-induced infectious disease–related pathways, such as the glyoxylate and dicarboxylate metabolism pathway and mTOR signaling pathway. A previous study revealed that inhibition of cellular mTORC1 was correlated with HP vacuolating cytotoxin (VacA)-dependent amino acid starvation (Kim I.-J. et al., 2018). Furthermore, metabolomics combined with transcriptomics analysis showed that the carbohydrate metabolism and citric acid might be downstream regulators of the function of metabolic genes in HP-induced GC. Recently, accumulating evidence has shown that citrate can act as a metabolic regulator and is involved in numerous physiological and pathophysiological processes, such as inflammation and cancer (Infantino et al., 2011; Icard et al., 2012). Therefore, our study sheds light on molecular targets and metabolites and pathways responsible for HP-induced GC from a metabolic perspective.

HP-associated dysbiosis of the gastric flora is of particular importance in research because studies have shown increased relative abundance of specific taxa in patients who develop premalignant lesions and GC (Aviles-Jimenez et al., 2014). More recently, an exploratory study showed a significant relationship between the gut microbiome and metabolome datasets by comparing HP+ and HP− patients (White et al., 2021). The results of a study on the intragastric microenvironment of gastric cancer patients showed that the frequency and abundance of HP were significantly lower in the cancer group than in the non-cancer group. And *Clostridium*, *Fusobacterium*, and *Lactobacillus* species were frequently abundant in the gastric cancer group (Hsieh et al., 2018). The 16sRNA analysis of aforementioned studies revealed a possible role of *Lactobacillus* in HP infection leading to gastric carcinogenesis. The metabolites of *Lactobacillus* are important raw materials for glycolysis itself, coinciding with our results (Figures 6G,H). In conclusion, we observed an important role of HP or non-HP in altering the microbial diversity of the gastrointestinal tract and an important impact on the gastrointestinal metabolism.

Regarding the previously reported relationship between drug resistance and tumor metabolic characteristics (Yoshida, 2015), we investigated whether the expression of the five MRGs could be associated with sensitivity to anticancer drugs. We demonstrated that the five MRGs were explicitly associated with sensitivity or resistance to chemotherapy. MRGs can be used as targets for the personalized treatment of HP-induced GC patients. In future studies, we intend to elucidate the pharmacological mechanisms of action of the five MRGs (GSS, GMPPA, OGDH, SGPP2, and PIK3CA) against HP-induced GC through the modulation of their expression.

As mentioned before, metabolic genes are related to immune dysfunction and inflammatory stress in HP-induced GC. Furthermore, preclinical reports have confirmed a correlation between gene mutations and the response or tolerance to immunotherapy (Burr et al., 2017; George et al., 2017). It is important to understand and identify the metabolic interplay in cancer cells and immune cells and discuss the therapeutic opportunities as a result of this interplay to define targets for cancer treatment. Our analysis clearly showed that the five MRGs were strongly associated with immunotherapy response, including PD-1 and CTLA-4, validating their predictive value. Our findings also demonstrated the activation of the mTOR signaling pathway in groups with high expression of the five MRGs in HP+ GC. This suggests that PI3K-AKT-mTOR inhibitors coupled with immune checkpoint blockade might be beneficial for HP+ GC patients. A recent report also indicated that combining therapeutic strategies involving PI3K-AKT-mTOR inhibition with checkpoint blockade would be effective (O'Donnell et al., 2018). In general, the findings of the current investigation suggest that HP infection affects the therapeutic response by influencing the activity of key metabolic pathways, such as the mTOR pathway, in the host.

In this study, we established and verified a reliable metabolic model for HP+ GC and systematically linked these metabolic patterns to the characteristics of tumor immune cell infiltration. This comprehensive analysis suggested that metabolic dysregulation due to HP infection provided an important basis for a better understanding of tumor immunomodulation. More extensively, the five metabolic genes, metabolites, and metabolism pathways identified in this study provide an interesting starting point for considering the metabolic differences between HP+ GC and HP− GC as new prognostic markers or potential therapeutic targets.

## Data Availability

The datasets presented in this study can be found in online repositories. The names of the repository/repositories and accession number(s) can be found in the article/Supplementary Material.

## Abbreviations

CTLA-4, cytotoxic T-lymphocyte–associated protein 4; EMT, epithelial–mesenchymal transition; GC, gastric cancer; GMPPA, GDP–mannose pyrophosphorylase A; GSS, glutathione synthetase; HP, *Helicobacter pylori*; HP-MRGs, *Helicobacter pylori*–associated metabolism-related genes; ICIs, immune checkpoint inhibitors; MSI, microsatellite instability; MRGs, metabolism-related genes; MSGs, metabolic signature genes; mTOR, mechanistic target of rapamycin kinase; OGDH, oxoglutarate dehydrogenase; OS, overall survival; PCA, principal component analysis; PIK3CA, phosphatidylinositol-4,5-bisphosphate 3-kinase catalytic subunit alpha; PD-L1, programmed cell death ligand 1; PD-1, programmed cell death-1; ROS, reactive oxygen species; SGPP2, sphingosine-1-phosphate phosphatase 2; TIICs, tumor-infiltrating immune cells; TIDE, Tumor Immune Dysfunction and Exclusion.
