# Supplementary figures and images for: Transcriptomic and Metabolomic Profiling in Helicobacter pylori–Induced Gastric Cancer Identified Prognosis- and Immunotherapy-Relevant Gene Signatures
Source: Front Cell Dev Biol. 2021 Dec 24;9:769409. doi: 10.3389/fcell.2021.769409 (PMC8740065; doi:10.3389/fcell.2021.769409)

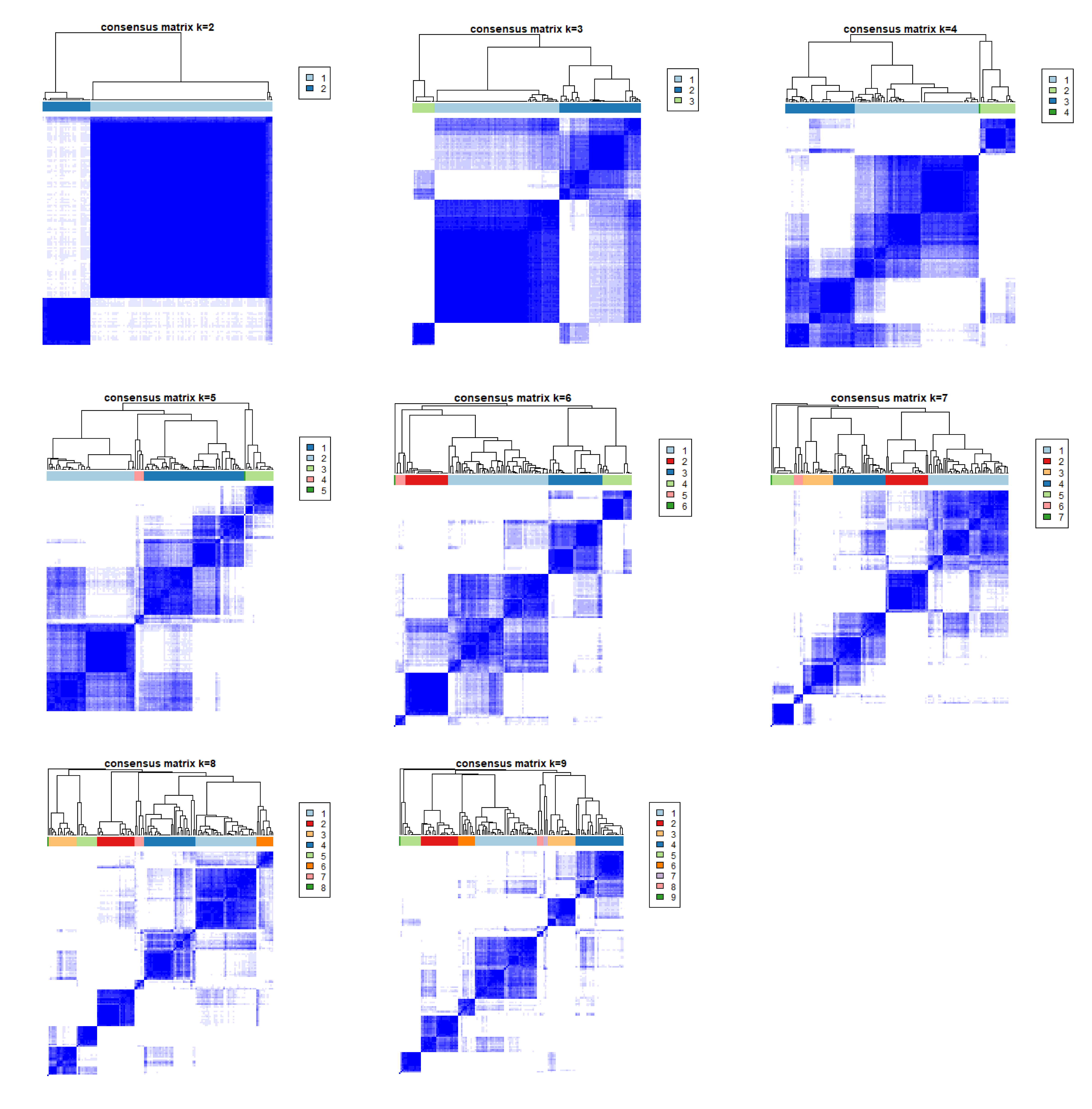

Supplement: Supplementary file 1 [file Image6.TIF]

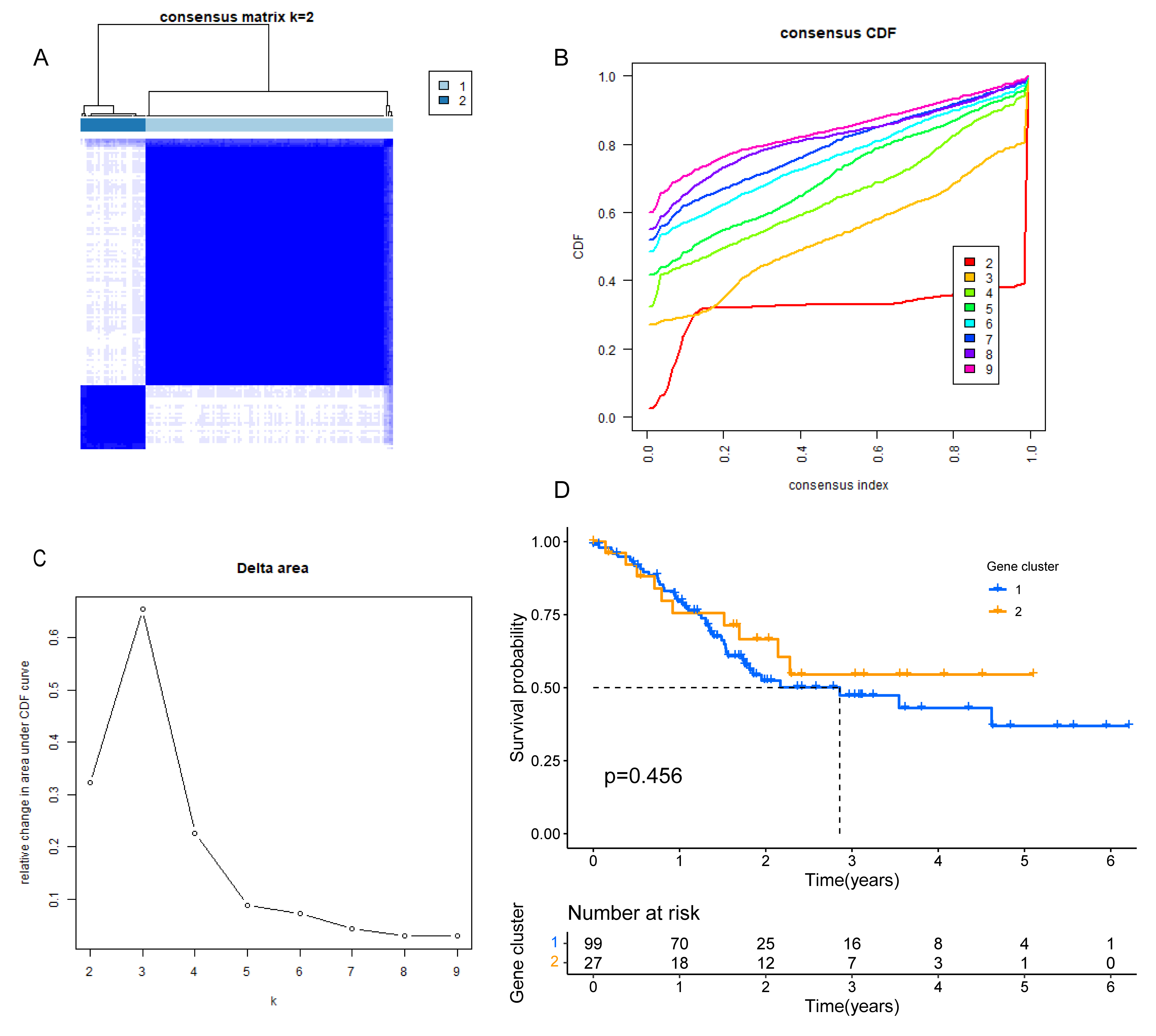

Supplement: Supplementary file 2 [file Image3.TIF]

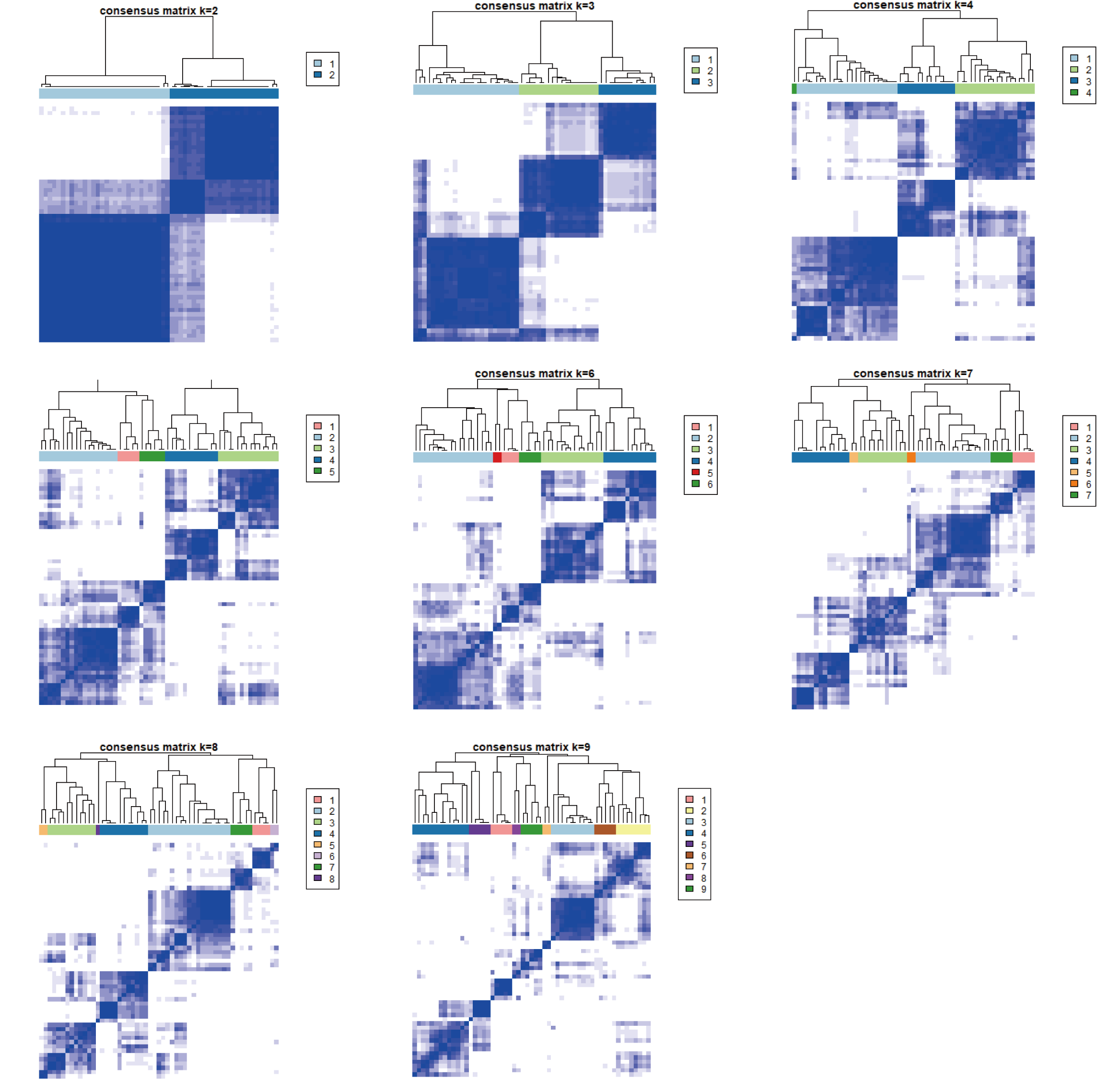

Supplement: Supplementary file 3 [file Image4.TIF]

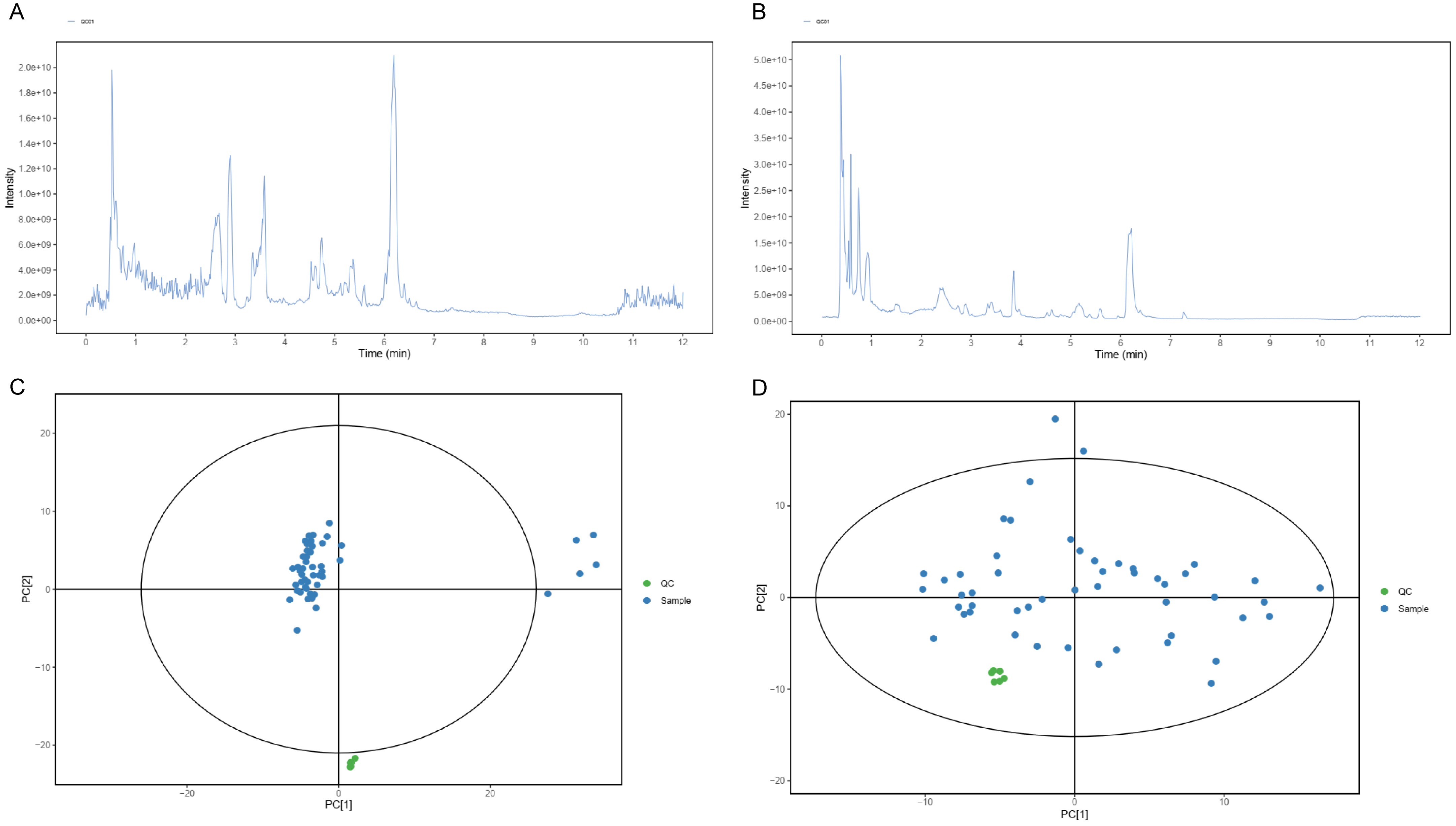

Supplement: Supplementary file 4 [file Image9.TIF]

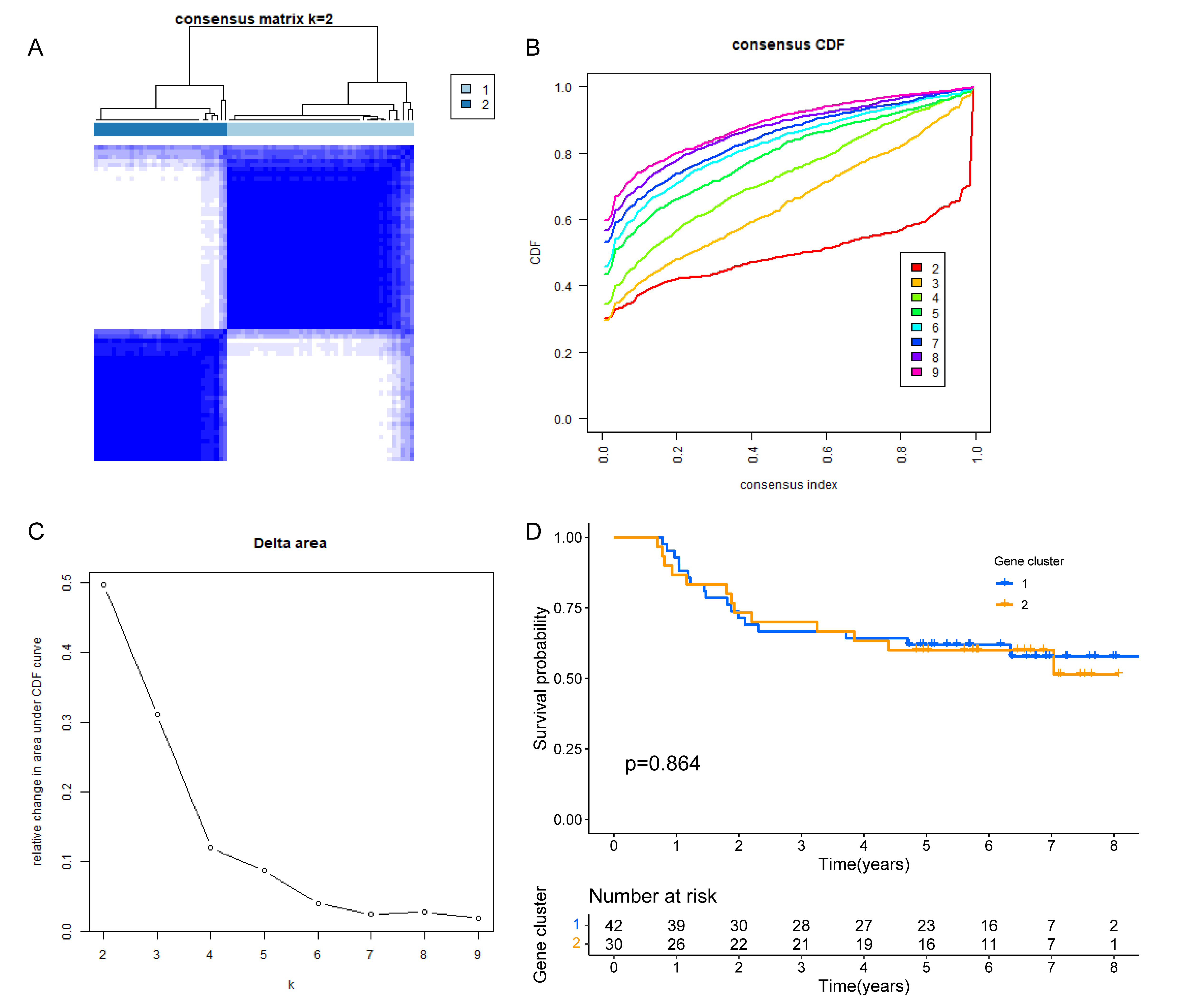

Supplement: Supplementary file 5 [file Image2.TIF]

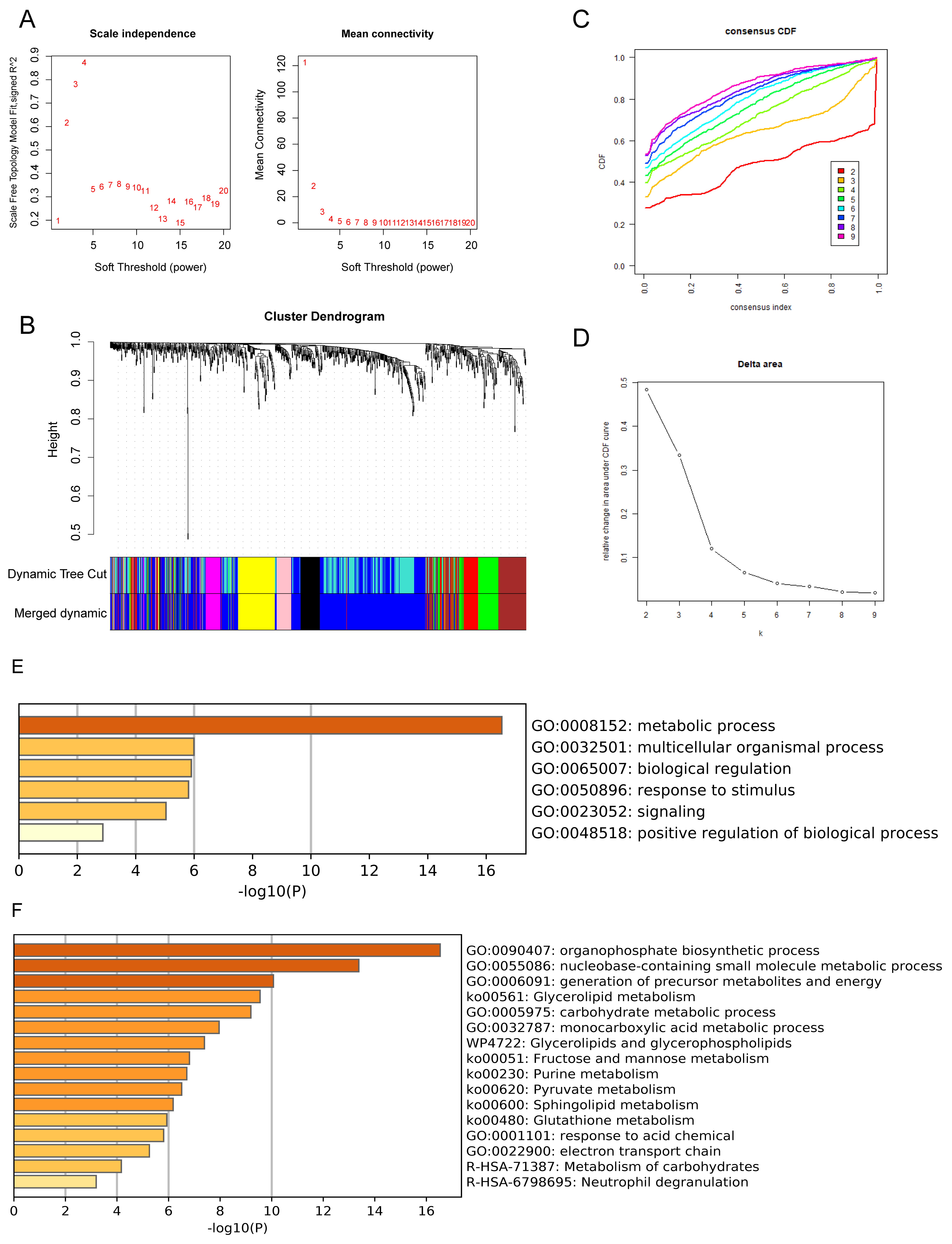

Supplement: Supplementary file 6 [file Image1.TIF]

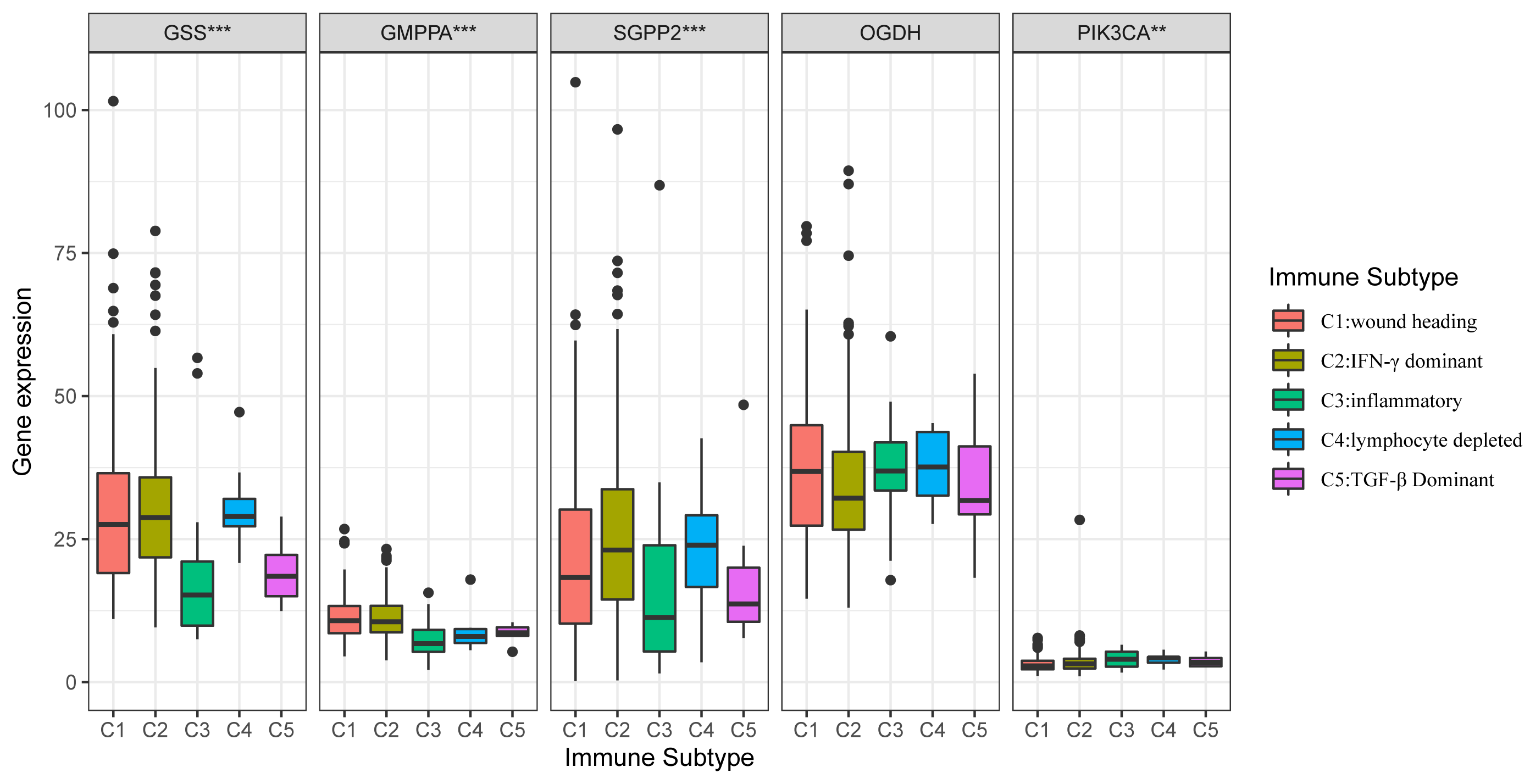

Supplement: Supplementary file 7 [file Image10.TIF]

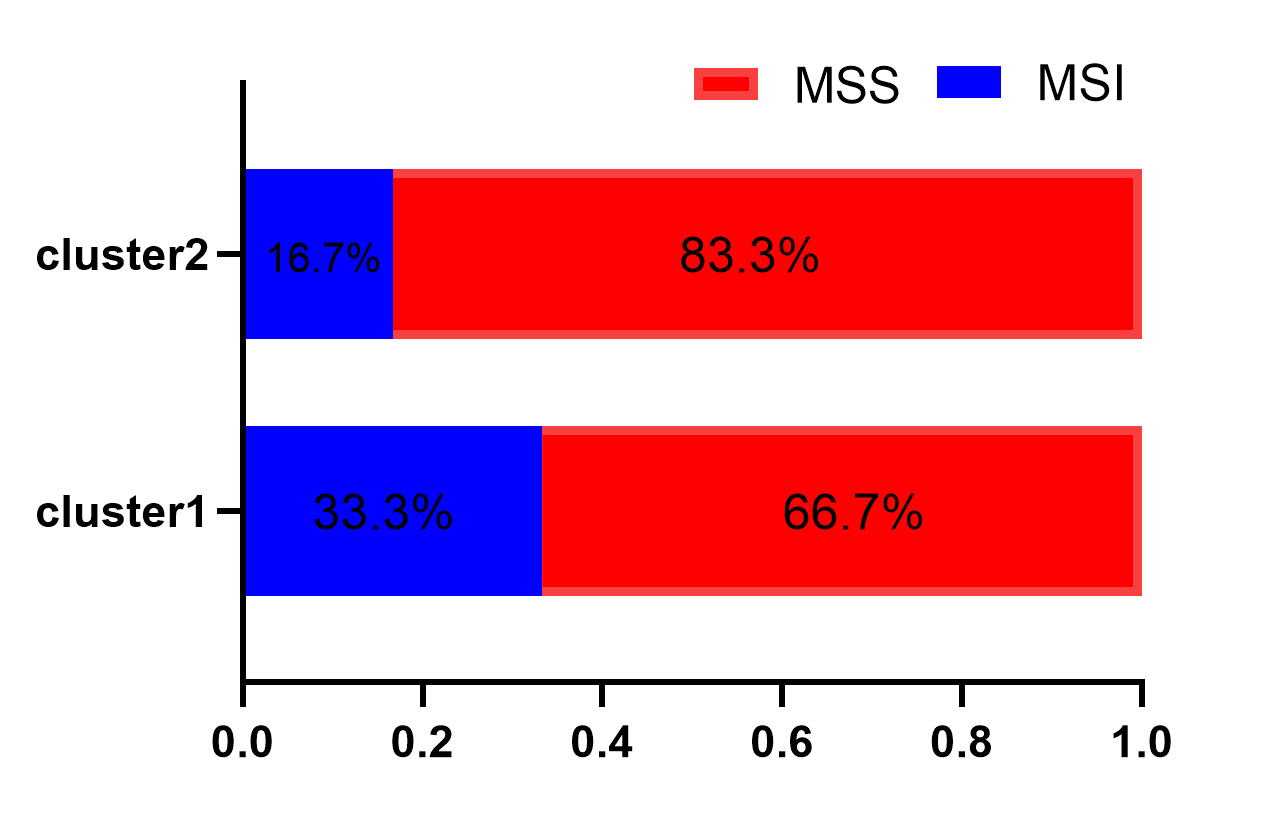

Supplement: Supplementary file 8 [file Image7.TIF]

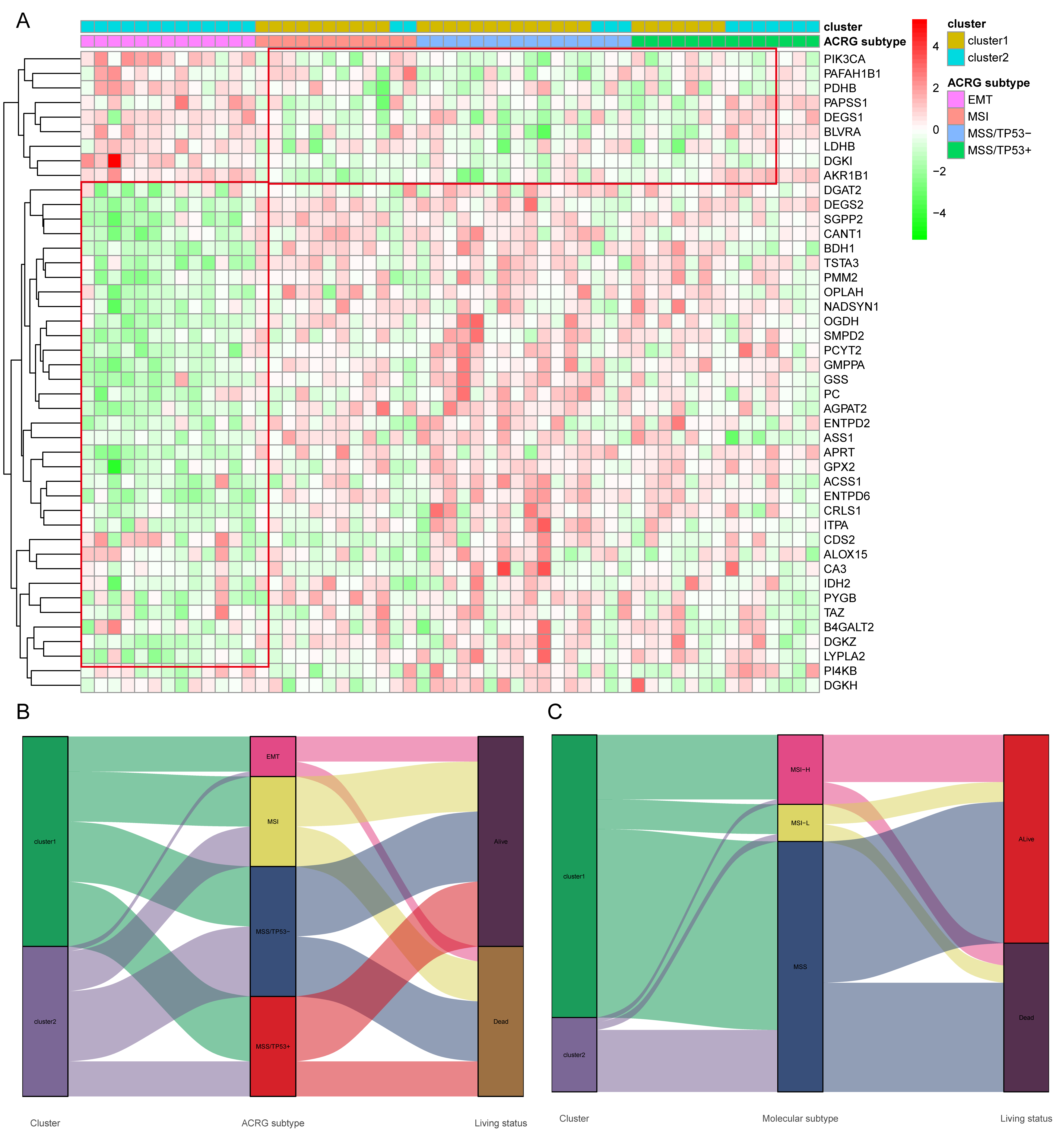

Supplement: Supplementary file 13 [file Image8.TIF]

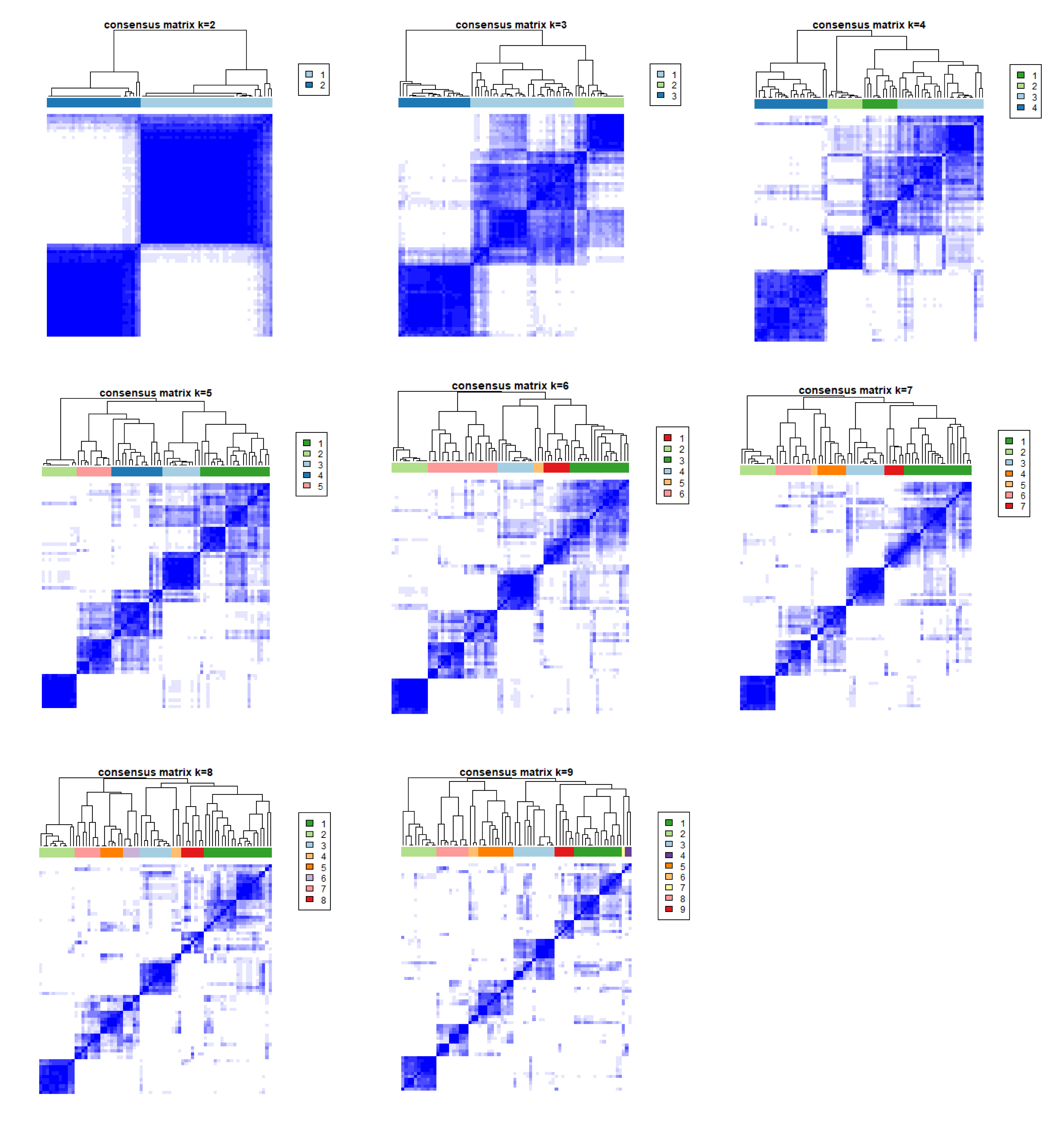

Supplement: Supplementary file 14 [file Image5.TIF]
