# Supplementary material for: Transcriptomic and Metabolomic Profiling in Helicobacter pylori–Induced Gastric Cancer Identified Prognosis- and Immunotherapy-Relevant Gene Signatures
Source: Front Cell Dev Biol. 2021 Dec 24;9:769409. doi: 10.3389/fcell.2021.769409 (PMC8740065; doi:10.3389/fcell.2021.769409)
Supplement: Supplementary file 9 [file Table2.DOCX]

**Table S2** Primers used for qRT-PCR

| Name | Sequence (5' -3') |
| --- | --- |
| GSS- Forward Primer | CTTCAACCTGCTAGTGGATGCTGT |
| GSS- Reverse Primer | TGGAACATGTAGTCTGAGCGATTC |
| GMPPA- Forward Primer | TGGAATGCAGGAGATTCTGCT |
| GMPPA- Reverse Primer | AGGATCTGGTCTCGAAAATGGTA |
| OGDH- Forward Primer | GGCTTCCCAGACTGTTAAGAC |
| OGDH- Reverse Primer | GCAGAATAGCACCGAATCTGTTG |
| SGPP2- Forward Primer | ACTGGAAAAGAGACTGATCGCT |
| SGPP2- Reverse Primer | CCTGCTGAGACACACCAAGG |
| PIK3CA-Forward Primer | CCACGACCATCATCAGGTGAA |
| PIK3CA- Reverse Primer | CCTCACGGAGGCATTCTAAAGT |
| β-Actin - Forward Primer | TTCCTTCCTGGGCATGGAGTCC |
| β-Actin - Reverse Primer | TGGCGTACAGGTCTTTGCGG |
