# Supplementary material for: Transcriptomic and Metabolomic Profiling in Helicobacter pylori–Induced Gastric Cancer Identified Prognosis- and Immunotherapy-Relevant Gene Signatures
Source: Front Cell Dev Biol. 2021 Dec 24;9:769409. doi: 10.3389/fcell.2021.769409 (PMC8740065; doi:10.3389/fcell.2021.769409)
Supplement: Supplementary file 10 [file Table3.DOCX]

Table S3 Clinical characteristics of included patients.

| Patient ID | Sex | Age(year) | Helicobacter pylori infection | Histopathological diagnosis |
| --- | --- | --- | --- | --- |
| GC-001 | Male | 65 | Infected | Gastric adenocarcinoma |
| GC-002 | Male | 66 | Infected | Gastric adenocarcinoma |
| GC-003 | Male | 65 | Infected | Gastric adenocarcinoma |
| GC-004 | Male | 62 | Infected | Gastric adenocarcinoma |
| GC-005 | Male | 56 | Infected | Gastric adenocarcinoma |
| GC-006 | Male | 50 | Infected | Gastric adenocarcinoma |
| GC-007 | Female | 47 | Uninfected | Gastric adenocarcinoma |
| GC-008 | Male | 49 | Uninfected | Gastric adenocarcinoma |
| GC-009 | Female | 55 | Uninfected | Gastric adenocarcinoma |
| GC-010 | Female | 57 | Uninfected | Gastric adenocarcinoma |
| GC-011 | Male | 54 | Uninfected | Gastric adenocarcinoma |
| GC-012 | Male | 45 | Uninfected | Gastric adenocarcinoma |
| GC-013 | Female | 70 | Uninfected | Gastric adenocarcinoma |
| GC-014 | Male | 47 | Uninfected | Gastric adenocarcinoma |
| GC-015 | Male | 57 | Uninfected | Gastric adenocarcinoma |
| GC-016 | Female | 49 | Uninfected | Gastric adenocarcinoma |
| GC-017 | Female | 41 | Uninfected | Gastric adenocarcinoma |
| GC-018 | Male | 68 | Uninfected | Gastric adenocarcinoma |
| GC-019 | Female | 65 | Uninfected | Gastric adenocarcinoma |
| GC-020 | Male | 52 | Uninfected | Gastric adenocarcinoma |
| GC-021 | Male | 54 | Uninfected | Gastric adenocarcinoma |
| GC-022 | Male | 64 | Uninfected | Gastric adenocarcinoma |
| GC-023 | Male | 66 | Uninfected | Gastric adenocarcinoma |
| GC-024 | Male | 66 | Uninfected | Gastric adenocarcinoma |
| GC-025 | Male | 62 | Uninfected | Gastric adenocarcinoma |
| WY-014 | Male | 49 | Uninfected | Non-atrophic gastritis |
| WY-015 | Male | 66 | Uninfected | Non-atrophic gastritis |
| WY-016 | Female | 80 | Uninfected | Non-atrophic gastritis |
| WY-017 | Male | 68 | Uninfected | Non-atrophic gastritis |
| WY-018 | Female | 61 | Uninfected | Non-atrophic gastritis |
| WY-019 | Male | 47 | Uninfected | Non-atrophic gastritis |
| WY-020 | Female | 52 | Uninfected | Non-atrophic gastritis |
| WY-021 | Male | 47 | Uninfected | Non-atrophic gastritis |
| WY-022 | Female | 76 | Uninfected | Non-atrophic gastritis |
| WY-023 | Female | 51 | Uninfected | Non-atrophic gastritis |
| WY-024 | Female | 61 | Uninfected | Non-atrophic gastritis |
| WY-025 | Female | 57 | Uninfected | Non-atrophic gastritis |
