# Supplementary material for: Transcriptomic and Metabolomic Profiling in Helicobacter pylori–Induced Gastric Cancer Identified Prognosis- and Immunotherapy-Relevant Gene Signatures
Source: Front Cell Dev Biol. 2021 Dec 24;9:769409. doi: 10.3389/fcell.2021.769409 (PMC8740065; doi:10.3389/fcell.2021.769409)
Supplement: Supplementary file 12 [file Table4.DOCX]

Table S4. Multivariate analysis of the correlation of clinical variables with overall survival in HP+ GC.

| Clinical factors | Variables | HR (95%CI) | P value |
| --- | --- | --- | --- |
| Sex | Male vs Female | 1.699 (0.702-4.109) | 0.239 |
| Age | ≤ 65 vs >65 (years old) | 0.995 (0.367-2.694) | 0.992 |
| T Stage | T1-2 vs T3-4 | 3.299 (1.071-10.167) | 0.038 |
| N Stage | N0 vs N1-3 | 1.835 (0.529-6.368) | 0.339 |
| M Stage | M0 vs M1 | 13.765 (3.494-54.236) | 0.0002 |
| Cluster | Cluster1 vs Cluster2 | 1.427 (0.586-3.471) | 0.433 |
| Metabolic score | High score vs Low score | 0.788 (0.272-2.285) | 0.661 |
